# Supplementary material for: Structure and quenching of a bundle-shaped phycobilisome
Source: Sci Adv. 2025 Oct 15;11(42):eadz6774. doi: 10.1126/sciadv.adz6774 (PMC12525769; doi:10.1126/sciadv.adz6774)
Supplement: Supplementary file 1 — Figs. S1 to S21 Tables S1 and S2 Legend for movie S1 References [file sciadv.adz6774_sm.pdf]

Science Advances
AAAS

Supplementary Materials for

Structure and quenching of a bundle-shaped phycobilisome

Anna D. Burtseva *et al.*

Corresponding author: Nikolai N. Sluchanko, nikolai.sluchanko@mail.ru

Sci. Adv. **11**, eadz6774 (2025)
DOI: 10.1126/sciadv.adz6774

The PDF file includes:

Figs. S1 to S21
Tables S1 and S2
Legend for movie S1
References

Other Supplementary Material for this manuscript includes the following:

Movie S1

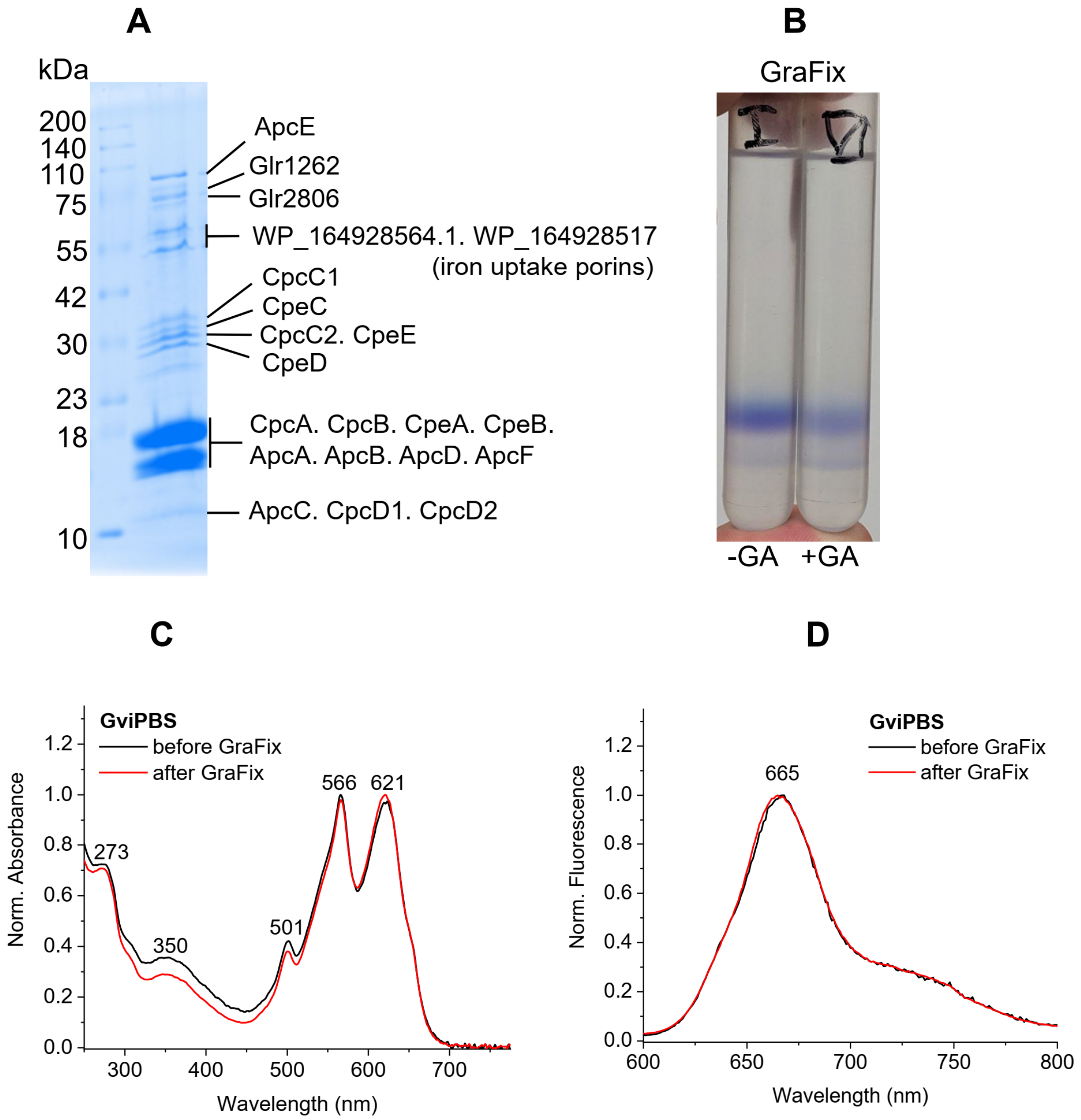

Fig. S1. *G. violaceus* PBS purification and characterization. A. SDS-PAGE analysis of the protein constituents of GviPBS. **B**. Ultracentrifugation tubes showing that the sedimentation pattern of GviPBS did not change in the presence of glutaraldehyde (GA) during GraFix (*15*). Absorbance (**C**) and fluorescence (**D**) spectra of the GviPBS sample obtained by ultracentrifugation before and after GraFix showing the preservation of the GviPBS assembly.

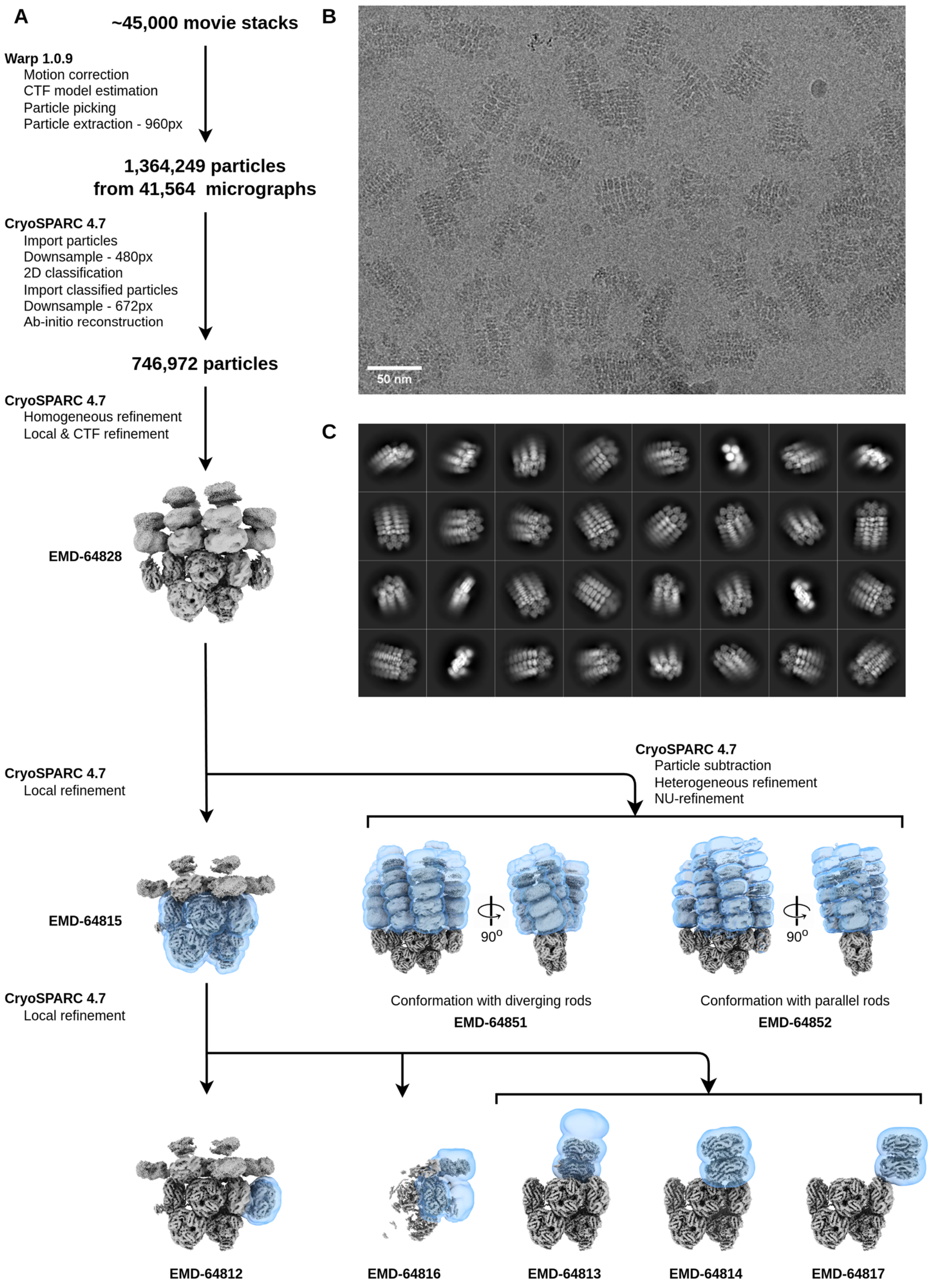

Fig. S2. Cryo-EM data processing workflow and 3D reconstruction. A. The workflow for the cryo-EM data processing. Cryo-EM density maps of GviPBS are shown in grey, masks used for the local refinement are shown in transparent blue. Each map is labeled with the corresponding EMDB code. **B**. A representative motion-corrected electron micrograph of GviPBS. The scale bar corresponds to 50 nm. **C**. 2D classification of the cryo-EM data demonstrating different views of the GviPBS.

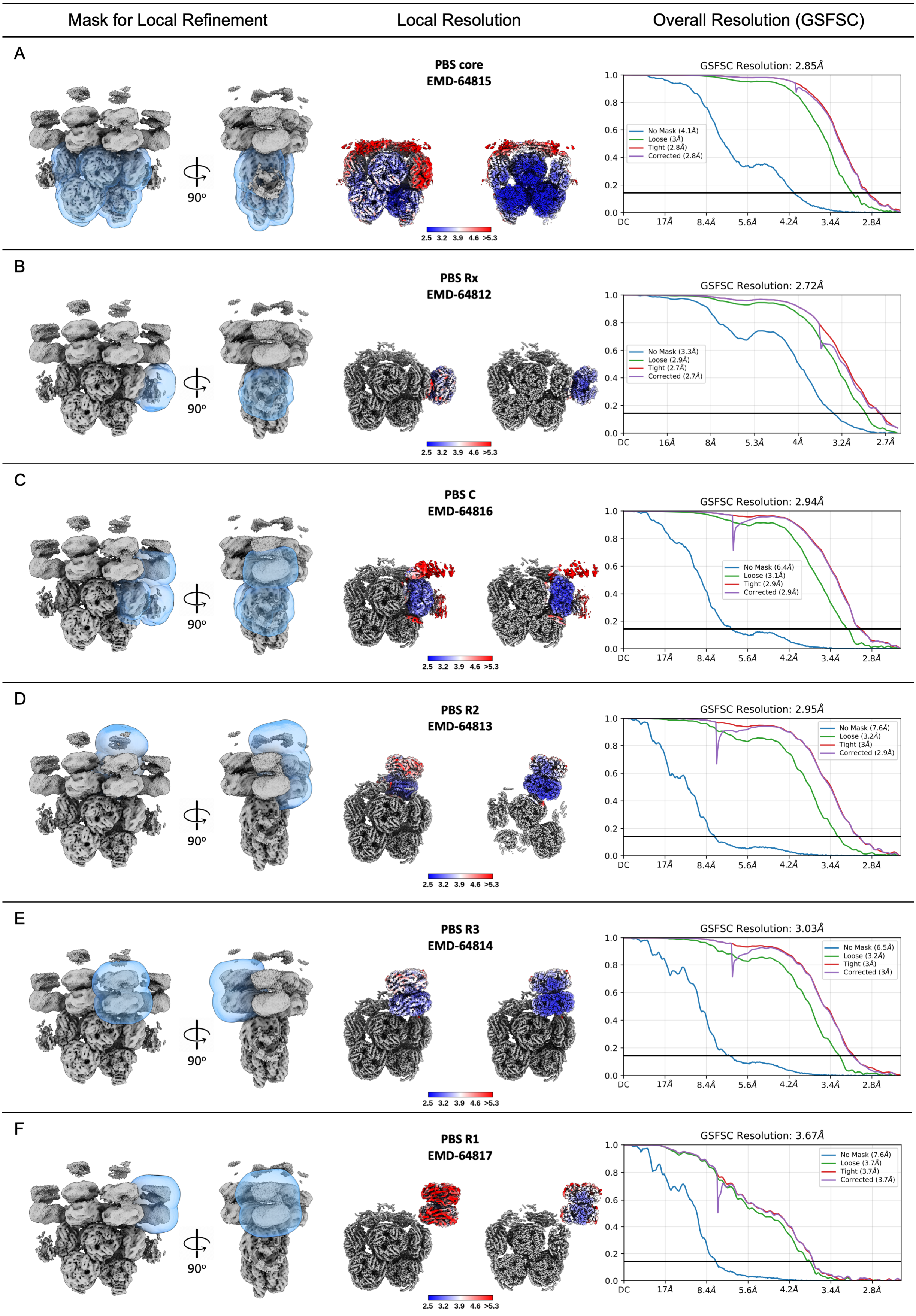

Mask for Local Refinement
Local Resolution
Overall Resolution (GSFSC)
A
90º
PBS core
EMD-64815
2.5 3.2 3.9 4.6 >5.3
GSFSC Resolution: 2.85Å
No Mask (4.1Å)
Loose (3Å)
Tight (2.8Å)
Corrected (2.8Å)
B
90º
PBS Rx
EMD-64812
2.5 3.2 3.9 4.6 >5.3
GSFSC Resolution: 2.72Å
No Mask (3.3Å)
Loose (2.9Å)
Tight (2.7Å)
Corrected (2.7Å)
C
90º
PBS C
EMD-64816
2.5 3.2 3.9 4.6 >5.3
GSFSC Resolution: 2.94Å
No Mask (6.4Å)
Loose (3.1Å)
Tight (2.9Å)
Corrected (2.9Å)
D
90º
PBS R2
EMD-64813
2.5 3.2 3.9 4.6 >5.3
GSFSC Resolution: 2.95Å
No Mask (7.6Å)
Loose (3.2Å)
Tight (3Å)
Corrected (2.9Å)
E
90º
PBS R3
EMD-64814
2.5 3.2 3.9 4.6 >5.3
GSFSC Resolution: 3.03Å
No Mask (6.5Å)
Loose (3.2Å)
Tight (3Å)
Corrected (3Å)
F
90º
PBS R1
EMD-64817
2.5 3.2 3.9 4.6 >5.3
GSFSC Resolution: 3.67Å
No Mask (7.6Å)
Loose (3.7Å)
Tight (3.7Å)
Corrected (3.7Å)

Fig. S3. Overall and local resolution estimation for each local refinement. A. The core. **B**. Rx substructure. **C**. C/C' cylinder. **D**. R2 rod, two lowest hexamers. **E**. R3 rod, two lowest hexamers. **F**. R1 rod, two lowest hexamers. Left column: The masks used for the local refinement. The consensus cryo-EM density map is shown as gray, and the mask used for local refinement is shown as transparent blue. Middle column: Local resolution estimation for corresponded parts of PBS. The consensus cryo-EM density map is shown as gray, the locally refined cryo-EM density maps are colored according to the local resolution calculated by cryoSPARC, the color key is in Angstroms. Right column: Gold-standard FSC plots for the locally refined reconstructions. The global resolutions were estimated by gold-standard FSC (threshold at 0.143).

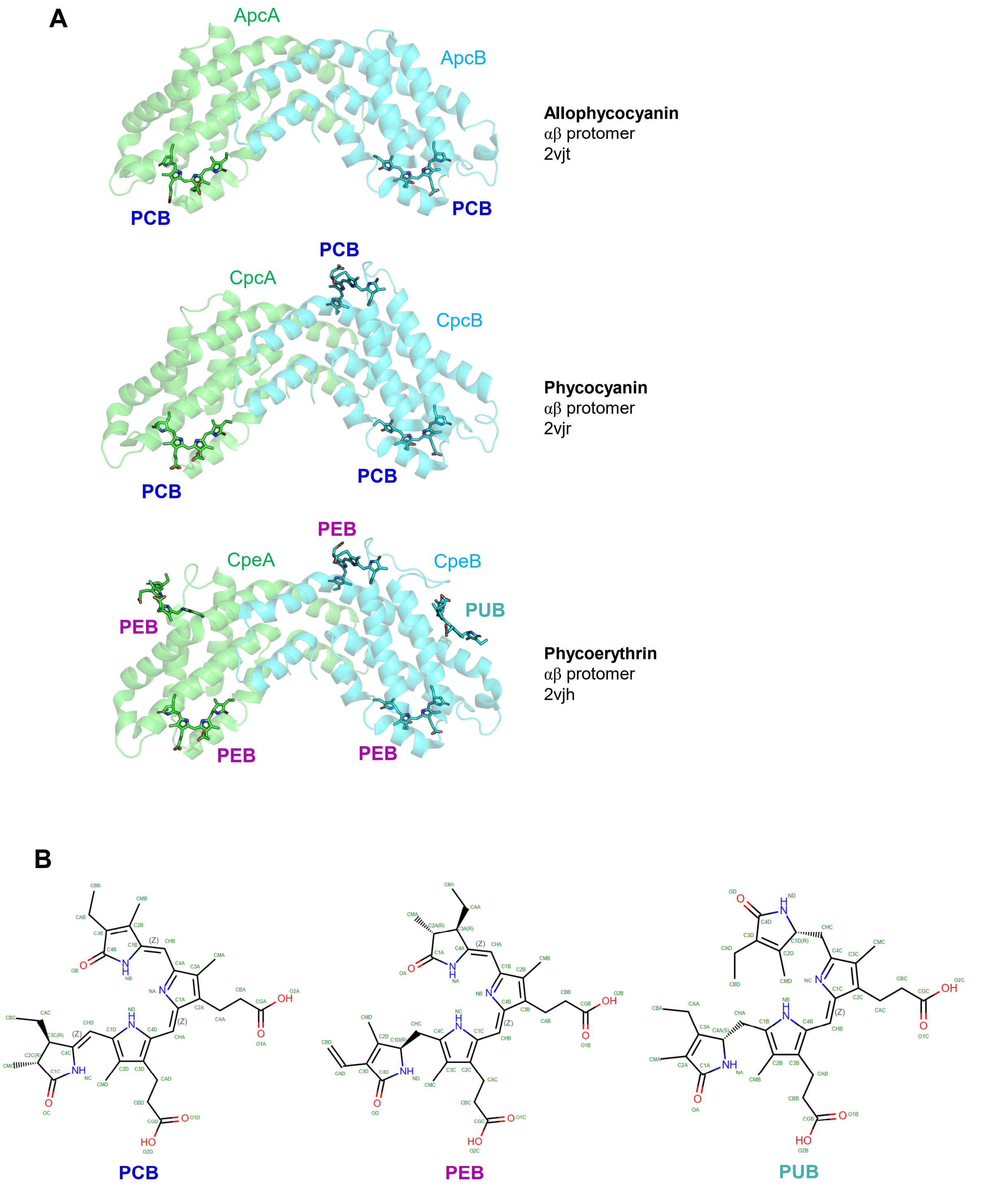

Fig. S4. Crystallographic structures of αβ protomers of allophycocyanin (AP), phycocyanin (PC) and phycoerythrin (PE) from *G. violaceus* with three main chromophores covalently attached to these PBPs. PCB – phycocyanobilin, PEB – phycoerythrobilin, PUB – phycourobilin. PDB codes are indicated. **A**. Cartoon representation of the PBP protomers with bilins shown as sticks. **B**. Chemical structures of the bilins from *G. violaceus* PBS.

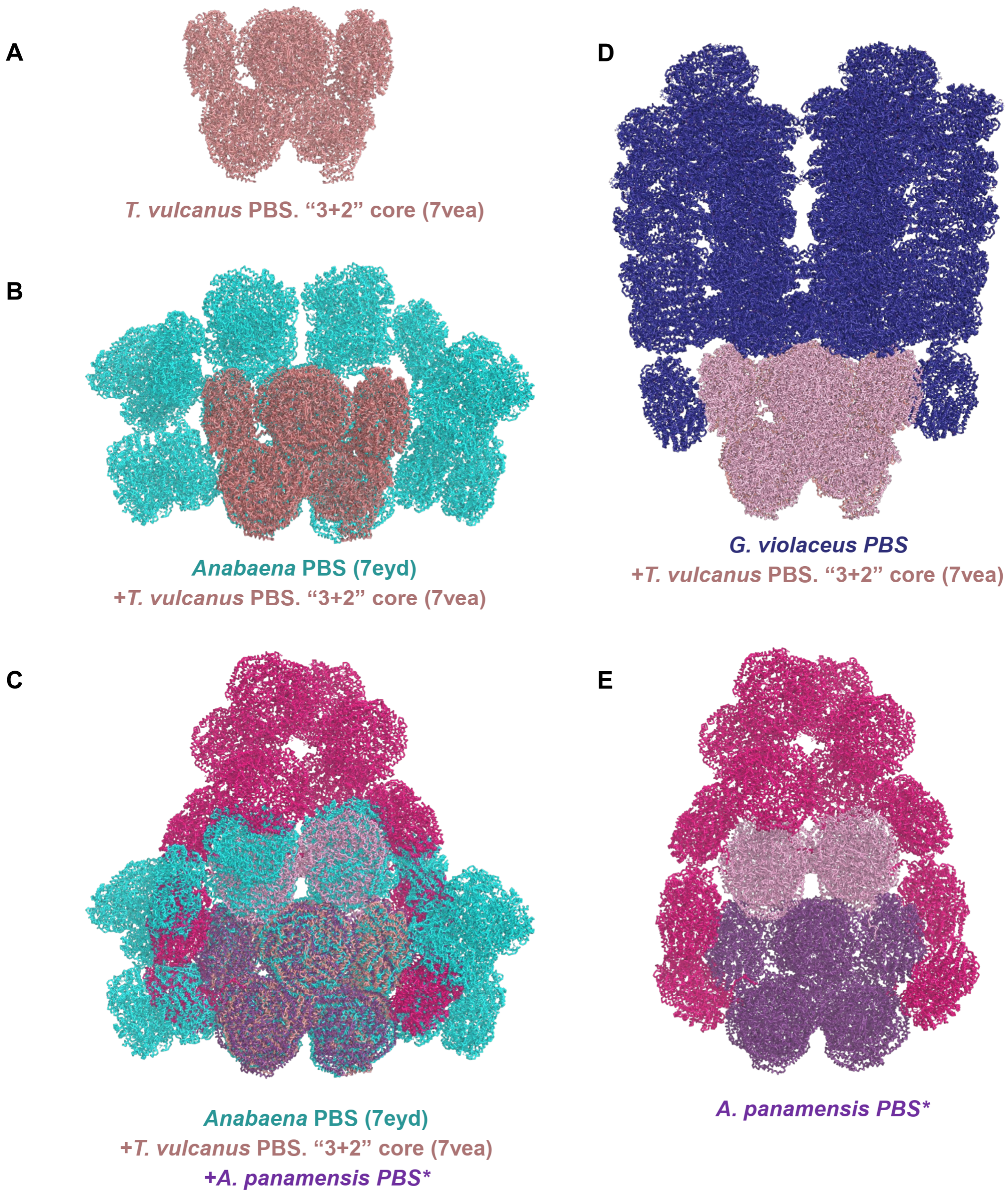

Fig. S5. The pentacylindrical core as the common substructure of PBS with different morphologies. A. The pentacylindrical core of *T. vulcanus* PBS. **B**. The pentacylindrical core of *Anabaena* PBS (*8*) shown overlaid with that of *T. vulcanus* (*17*). **C**. Overlay of the structures from panel B with the structure of the paddle-shaped PBS from *A. panamensis* (*5*). **D**. GviPBS with the pentacylindrical core (this work). **E**. The paddle-shaped PBS from *A. panamensis* (*5*).

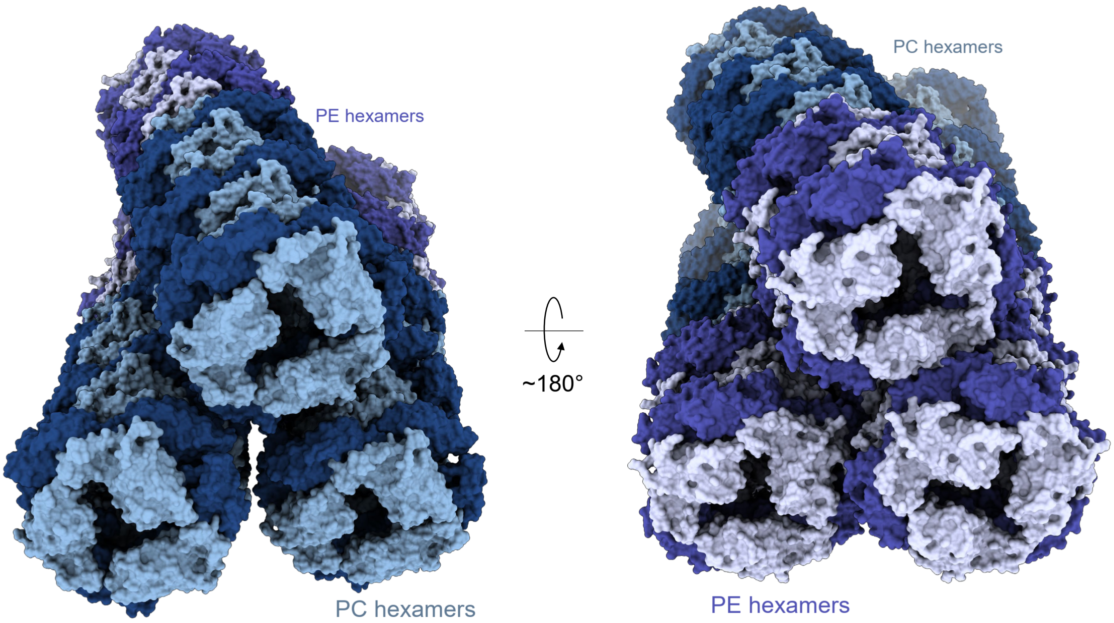

Fig. S6. Helicity of rod bundles in GviPBS. Alpha and beta PBP subunits are shown by dark and light tints of color, respectively. Two views of a rod bundle showing non-parallel and helical orientation of the rods. The period of a superhelix is estimated as ~1000 Å.

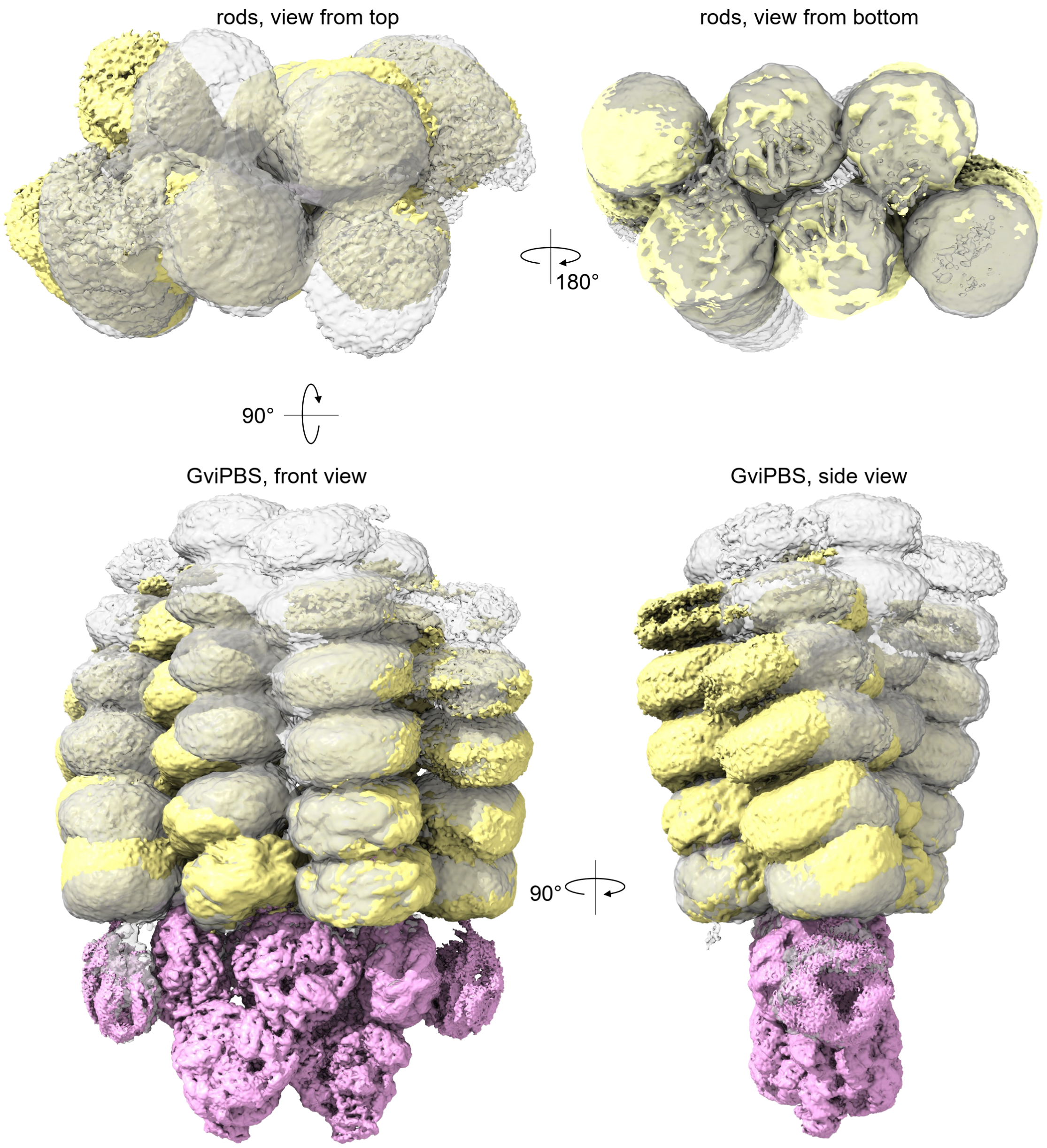

Fig. S7. Conformational heterogeneity in rod bundles in GviPBS. Shown are different views of the superposition of two cryo-EM maps (light yellow and grey) corresponding to the two different conformational states of the rod bundles. Pink map corresponds to the AP core with Rx1/Rx1' hexamers of PC; this map is omitted on the top views for clarity.

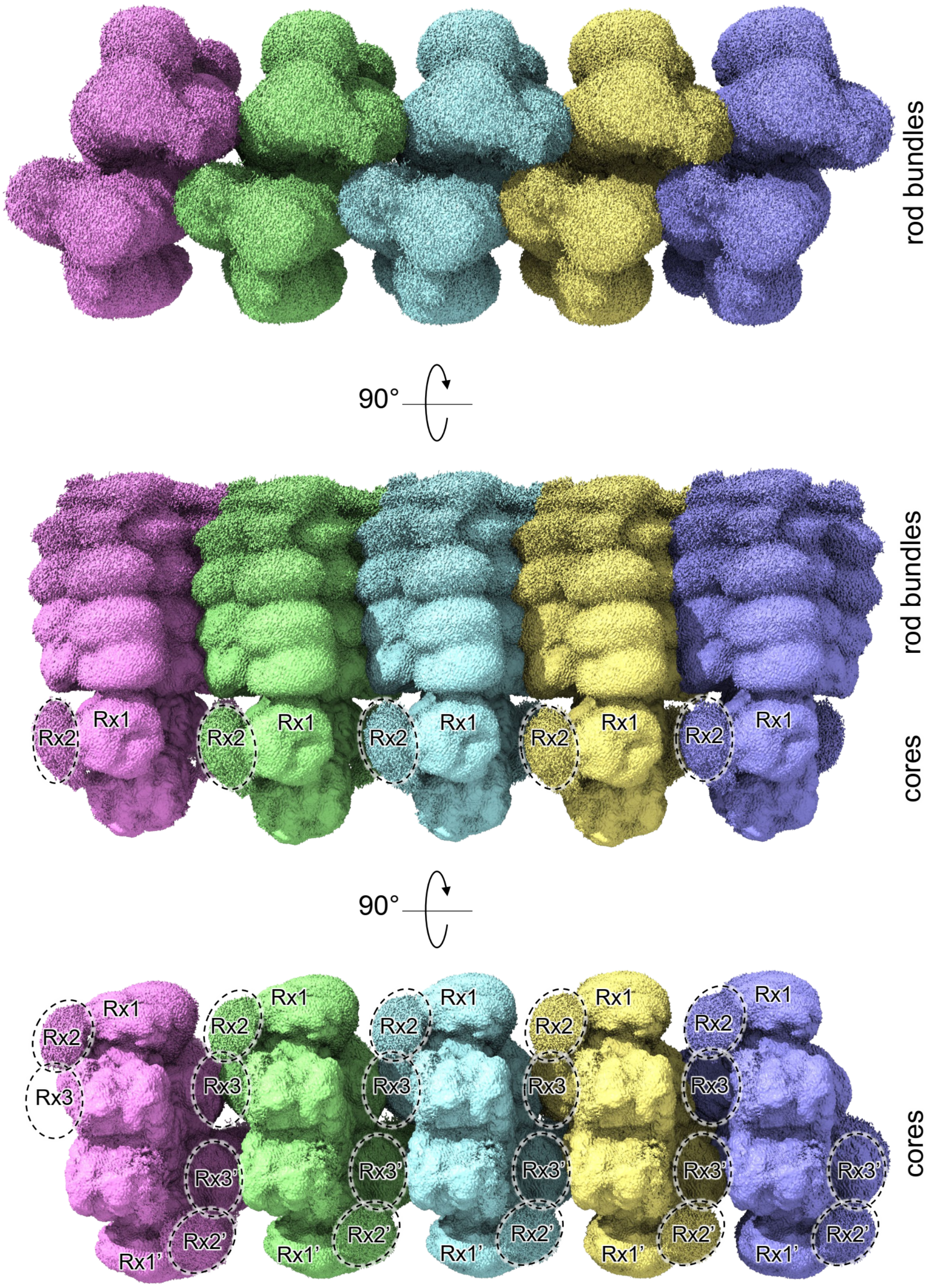

Fig. S8. Tentative model showing the principle of GviPBS assembly into arrays. Five stacked copies of the GviPBS cryo-EM map are shown from different views. Note that the poorly resolved density in the vicinity of Rx1/Rx1' suggests the position of the Rx2/Rx2' hexamers. The third hexamers Rx3/Rx3' are too dynamic and have no density, although their approximate location can be predicted from the presence of the third REP domain in the Glr2806 rod-core linkers (*16*). The complete GviPBS assemblies with six Rx hexamers (Rx1/Rx1', Rx2/Rx2', Rx3/Rx3') belting the AP core can attach to each other in arrays, which would effectively use limited space in the plasma membrane in thylakoid-less *G. violaceus* cells, consistent with earlier considerations and observations (*1*, *12*).

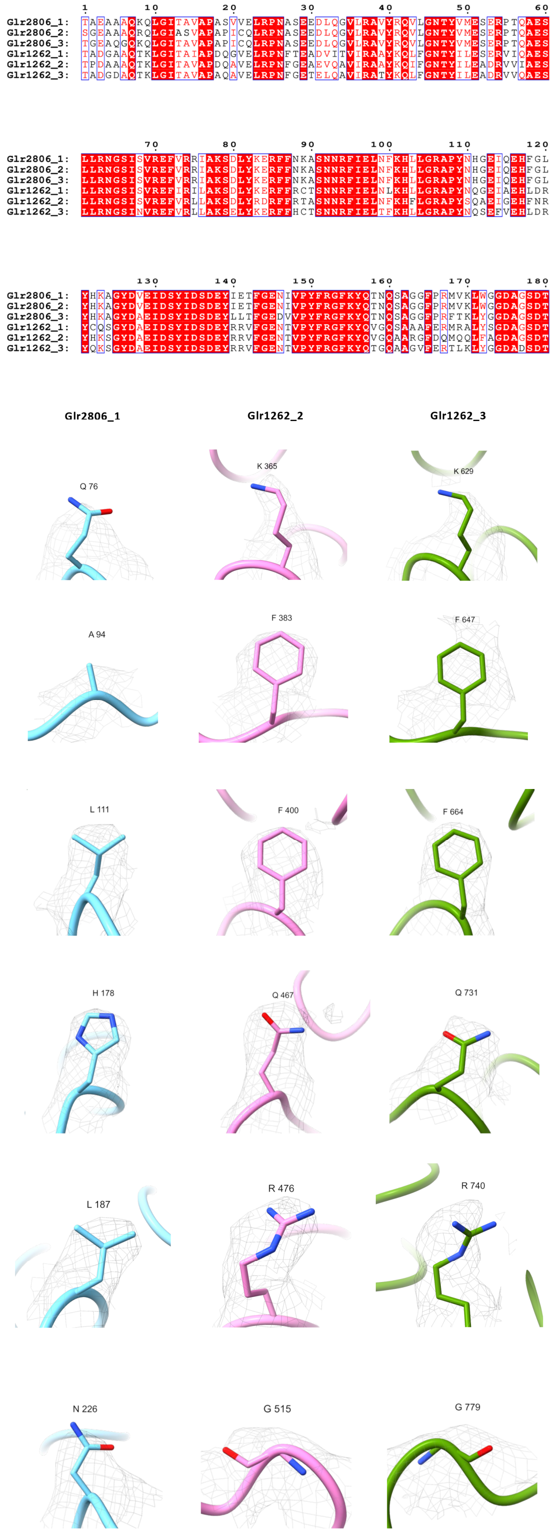

Fig. S9. Identification of Glr1262 and Glr2806 linkers by cryo-EM density for the positions specifically conserved only within REP domains of either protein. Top, multiple sequence alignment of REP domains of the two proteins. Bottom, representative demarcating positions supported by cryo-EM density.

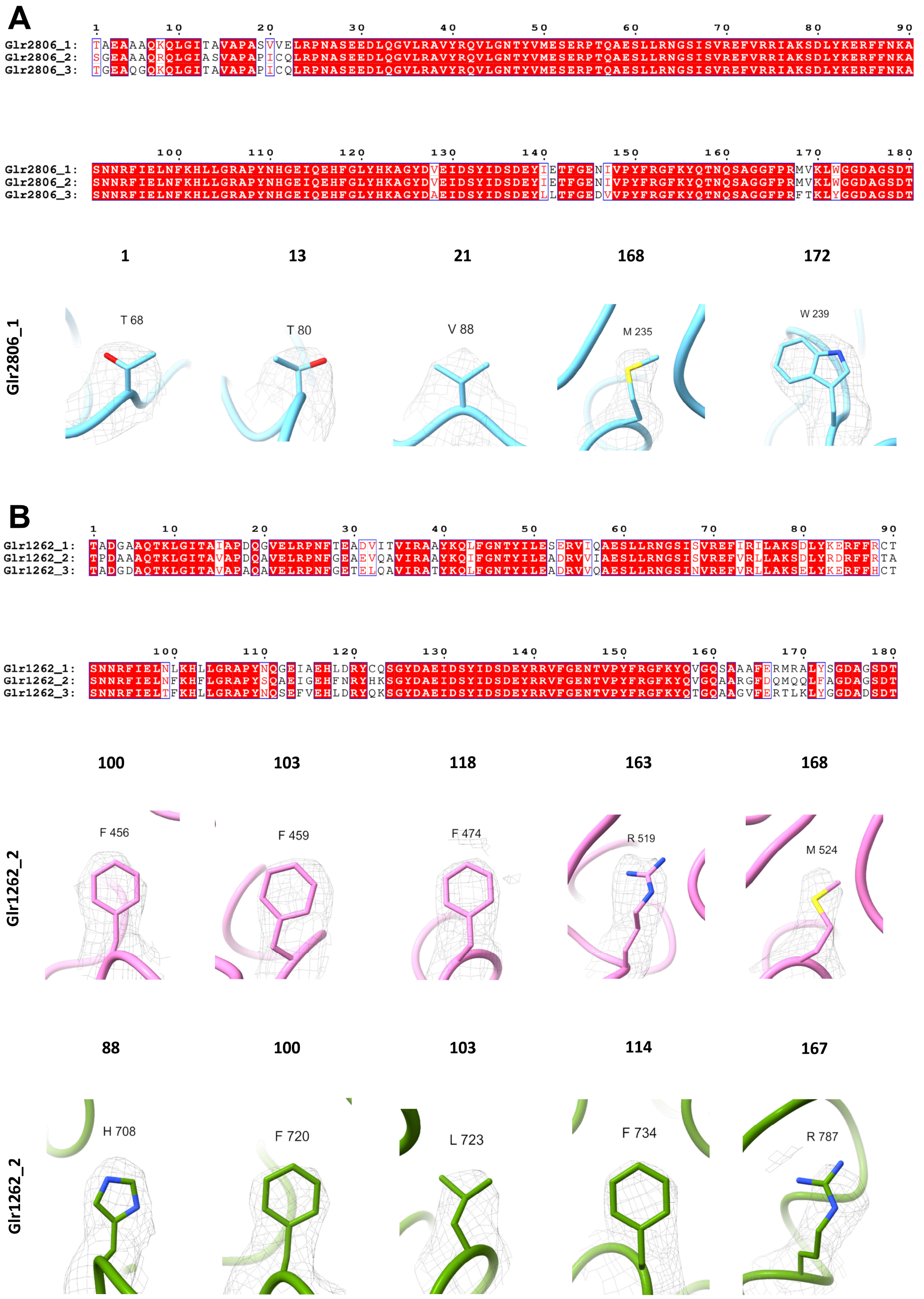

Fig. S10. Identification of REP domains within either Glr1262 or Glr2806 linkers by cryo-EM density for the nonconserved positions. A. Top, multiple sequence alignment of REP domains of Glr2806. Bottom, representative demarcating positions supported by cryo-EM density. **B**. Top, multiple sequence alignment of REP domains of Glr1262. Bottom, representative demarcating positions supported by cryo-EM density.

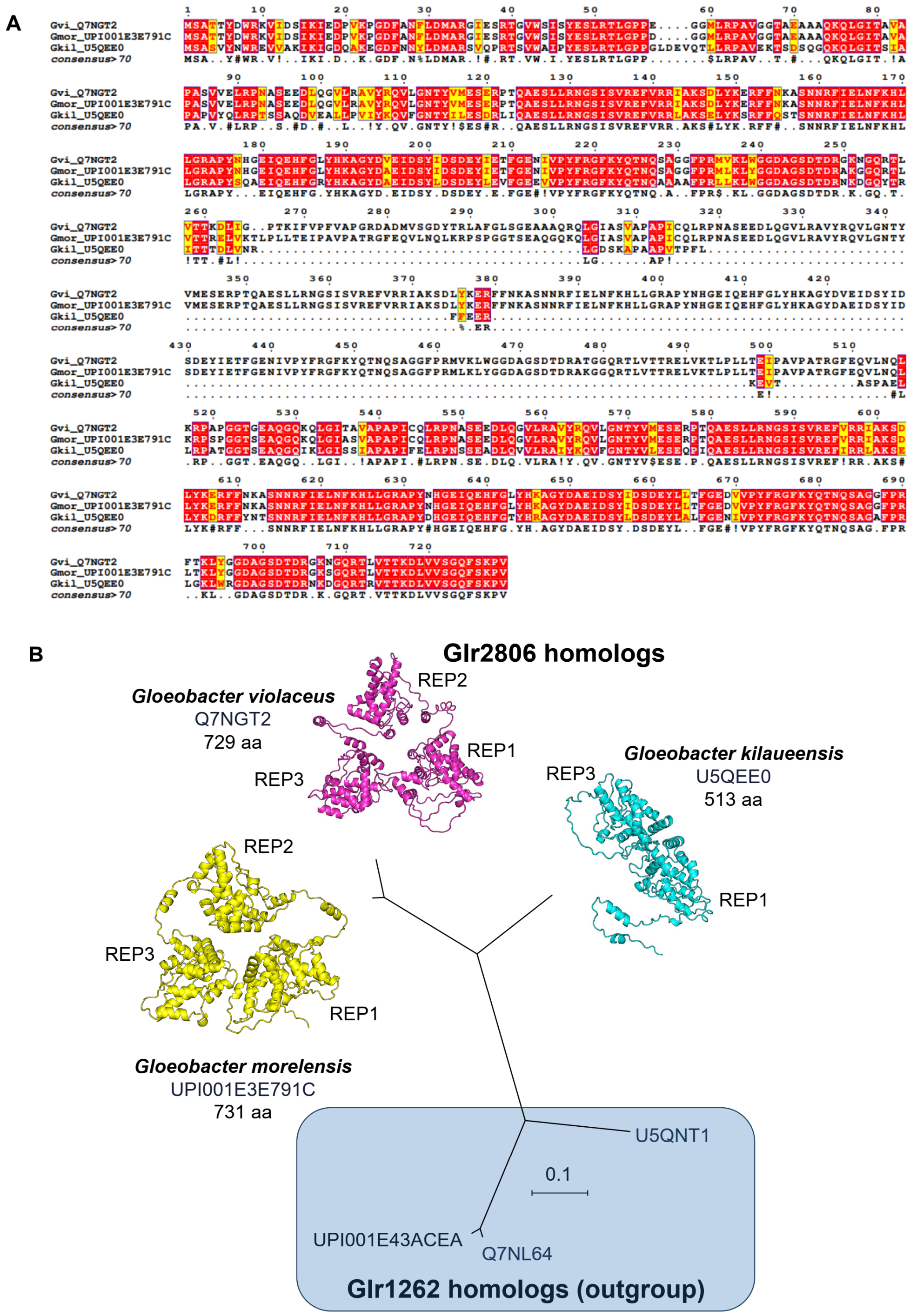

Fig. S11. Analysis of the number of REP domains in Glr2806 linkers of *Gloeobacter* species. **A**. Multiple sequence alignment of the Glr2806 homologs from *G. violaceus*, *G. morelensis* and *G. kilaueensis* showing that the latter has only two REP domains out of three found in other two homologs. **B**. Phylogenetic tree of Glr2806 homologs built using Glr1262 homologs from the same organisms as an outgroup. Scale bar represents the number of per residue substitutions. Alphafold3 (*58*) models of the three Glr2806 homologs analyzed are shown along with the corresponding REP domains numbered.

A

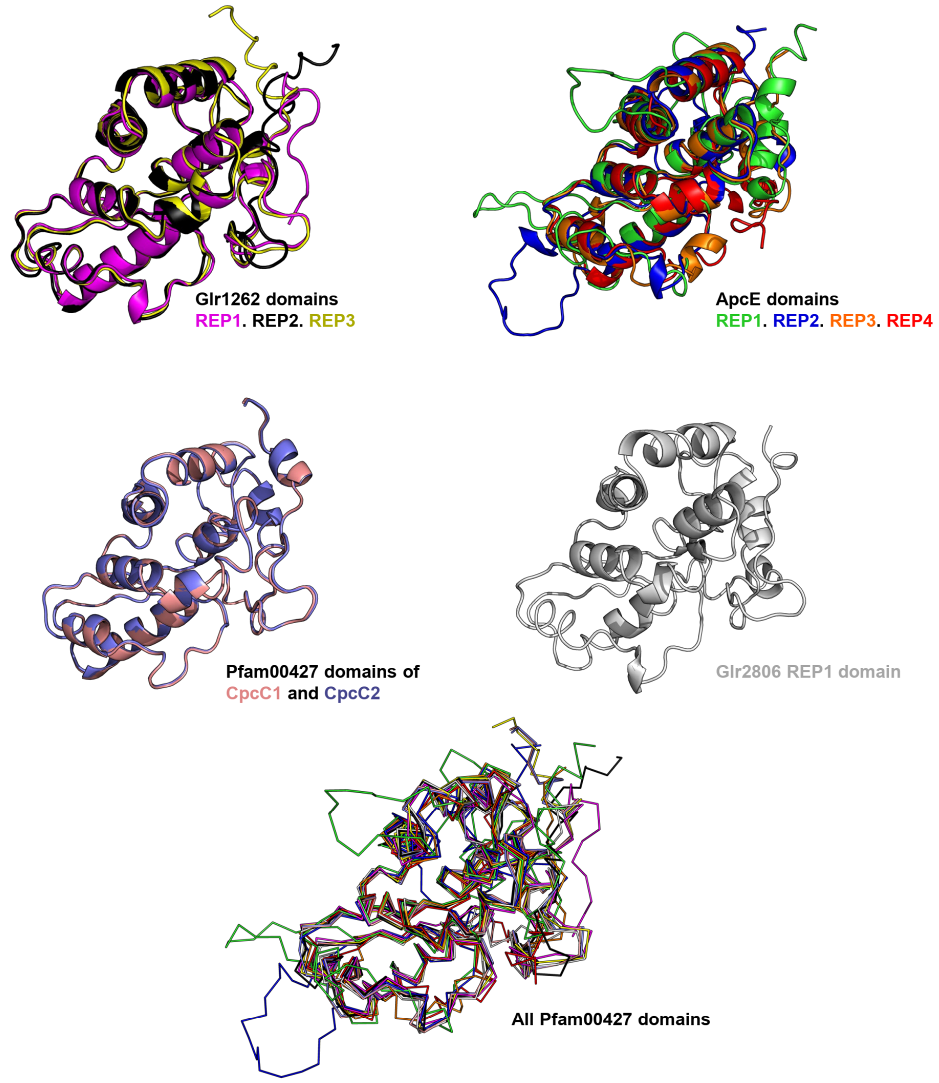

B

	Glr2806 REP1	Glr1262 REP1	Glr1262 REP2	Glr1262 REP3	ApcE REP1	ApcE REP2	ApcE REP3	ApcE REP4	CpcC1	CpcC2
Glr2806 REP1	x	1.034 (178)	0.624 (166)	0.698 (179)	1.34 (114)	1.124 (126)	0.936 (115)	1.169 (130)	0.794 (163)	0.827 (165)
Glr1262 REP1		x	0.813 (173)	0.657 (181)	1.749 (126)	1.045 (97)	0.803 (95)	0.928 (114)	1.002 (159)	0.934 (155)
Glr1262 REP2			x	0.498 (165)	1.552 (116)	1.009 (121)	0.928 (98)	1.006 (110)	0.801 (155)	0.755 (149)
Glr1262 REP3				x	1.117 (104)	0.987 (107)	0.846 (106)	1.094 (126)	0.832 (172)	0.756 (163)
ApcE REP1					x	1.149 (107)	0.743 (101)	0.963 (103)	1.636 (116)	1.680 (117)
ApcE REP2						x	0.764 (118)	0.833 (104)	1.136 (102)	1.168 (104)
ApcE REP3							x	0.741 (126)	1.002 (108)	0.999 (108)
ApcE REP4								x	0.934 (104)	0.888 (102)
CpcC1									x	0.196 (179)
CpcC2										x

Fig. S12. Pfam00427 domains of GviPBS linker proteins. A. Structural alignment of three REP domains of Glr1262, ApcE, CpcC1/CpcC2 and Glr2806 proteins. **B**. Cα RMSD values characterizing the similarity of the Pfam00427 domains found in GviPBS proteins. The values in Å were calculated upon structural alignment of a given domain pair. Numbers in parentheses indicate the number of the aligned atoms.

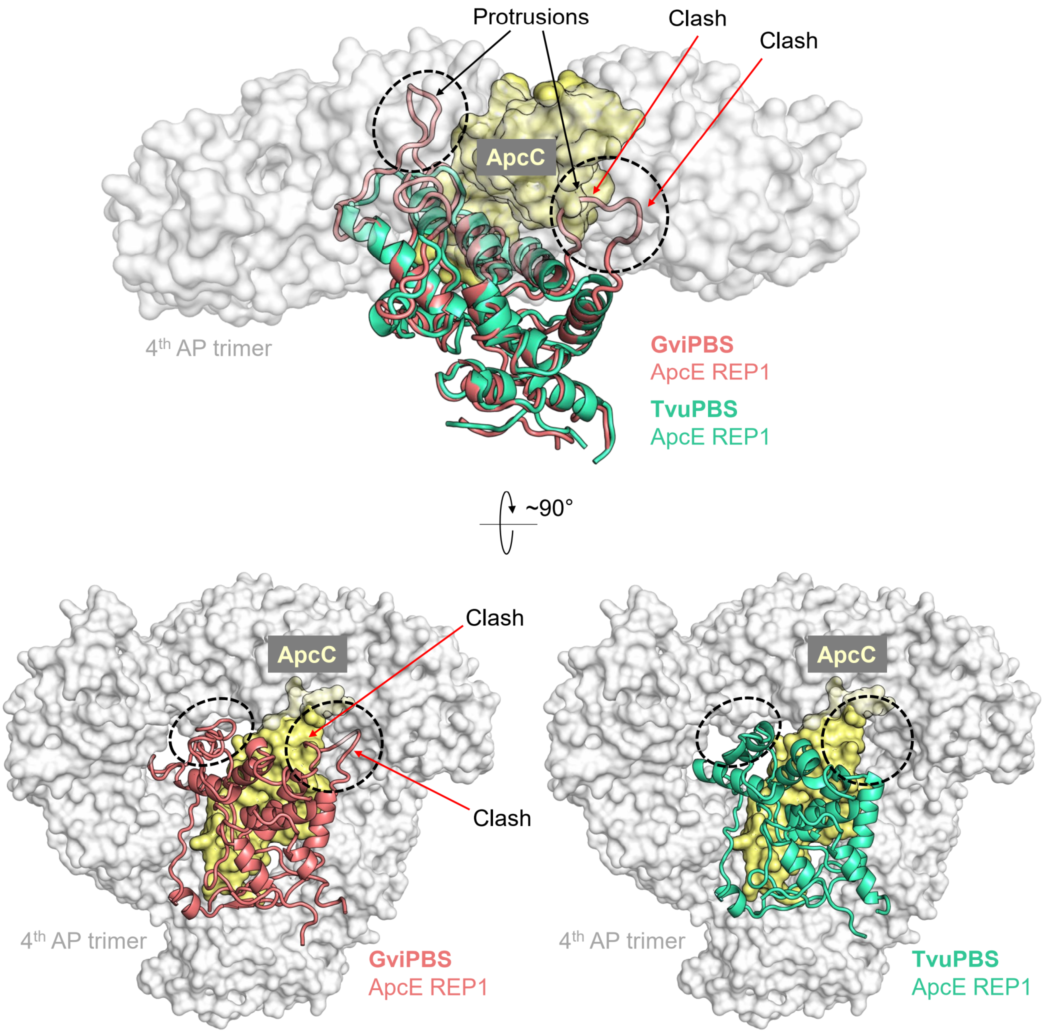

Fig. S13. Protrusions in ApcE of GviPBS interfere with the attachment of the ApcD-containing fourth trimer and ApcC. ApcE from GviPBS and PBS from *Thermosynechococcus vulcanus* (TvuPBS, PDB 7vea) were aligned to ApcE from *Anabaena* PBS that has the complete AP core with the fourth AP trimer containing ApcD (PDB 7eyd). This suggests a probable orientation of the fourth AP trimer relative to the REP1 domains of ApcE from GviPBS and TvuPBS, although this trimer is missing in both structures. Capping ApcC linker protein is also shown. As ApcE from GviPBS uniquely features the unstructured protrusions missing in ApcE from TvuPBS and other known ApcE proteins, those protrusions are likely to moderately interfere with the placement of the fourth AP trimer and ApcC. Although steric clashes (indicated by dashed circles and red arrows) are superficial, the unstructured protrusions likely sample larger space, thereby interfering with the attachment of the fourth trimer.

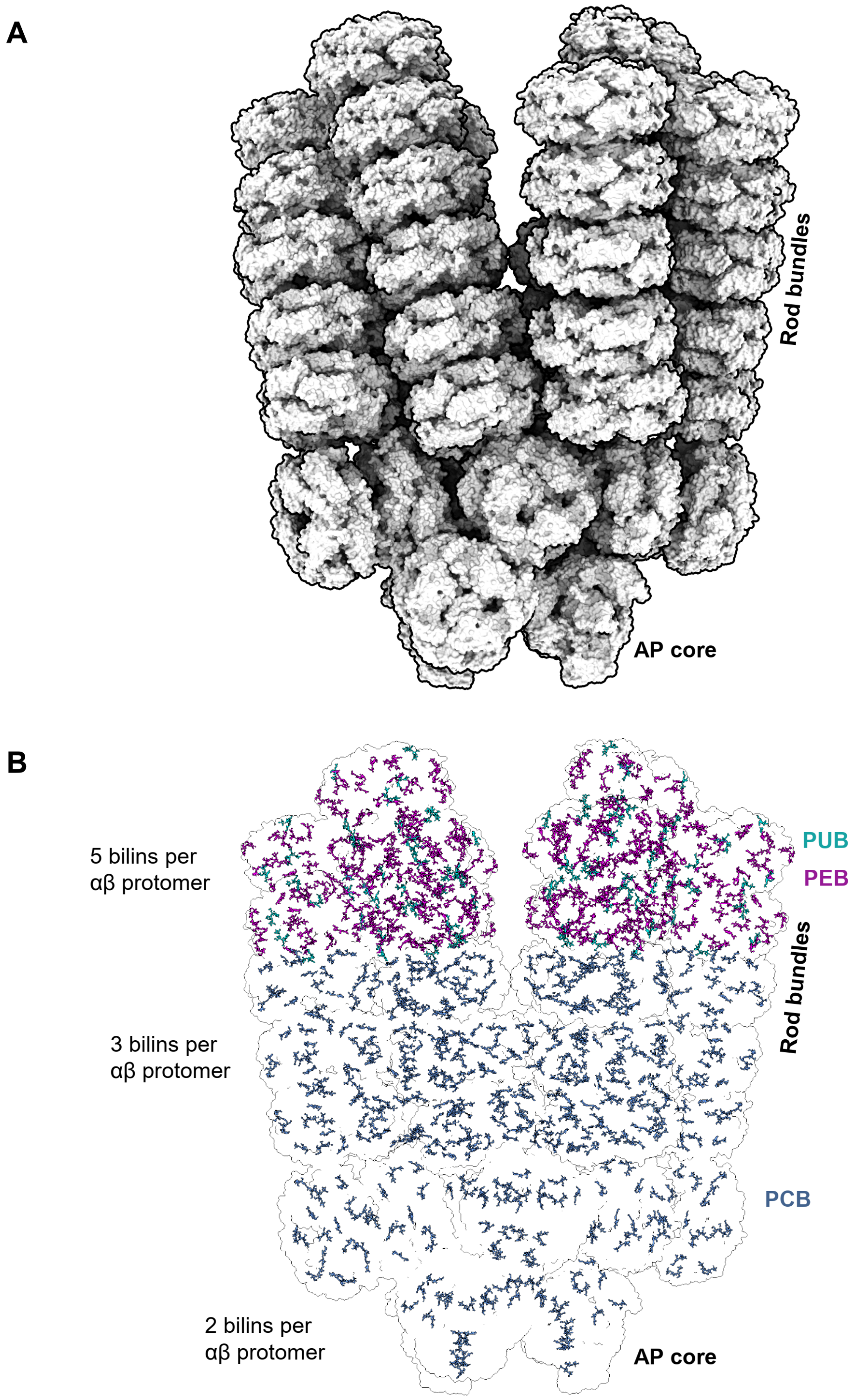

Fig. S14. GviPBS bilins exhibit a descending concentration gradient from the rods to the core. A. GviPBS model in surface representation. **B**. Silhouette of the GviPBS model showing the location of PCB, PEB and PUB. Given the different numbers of bilins bound in PE, PC and AP, the gradient is formed.

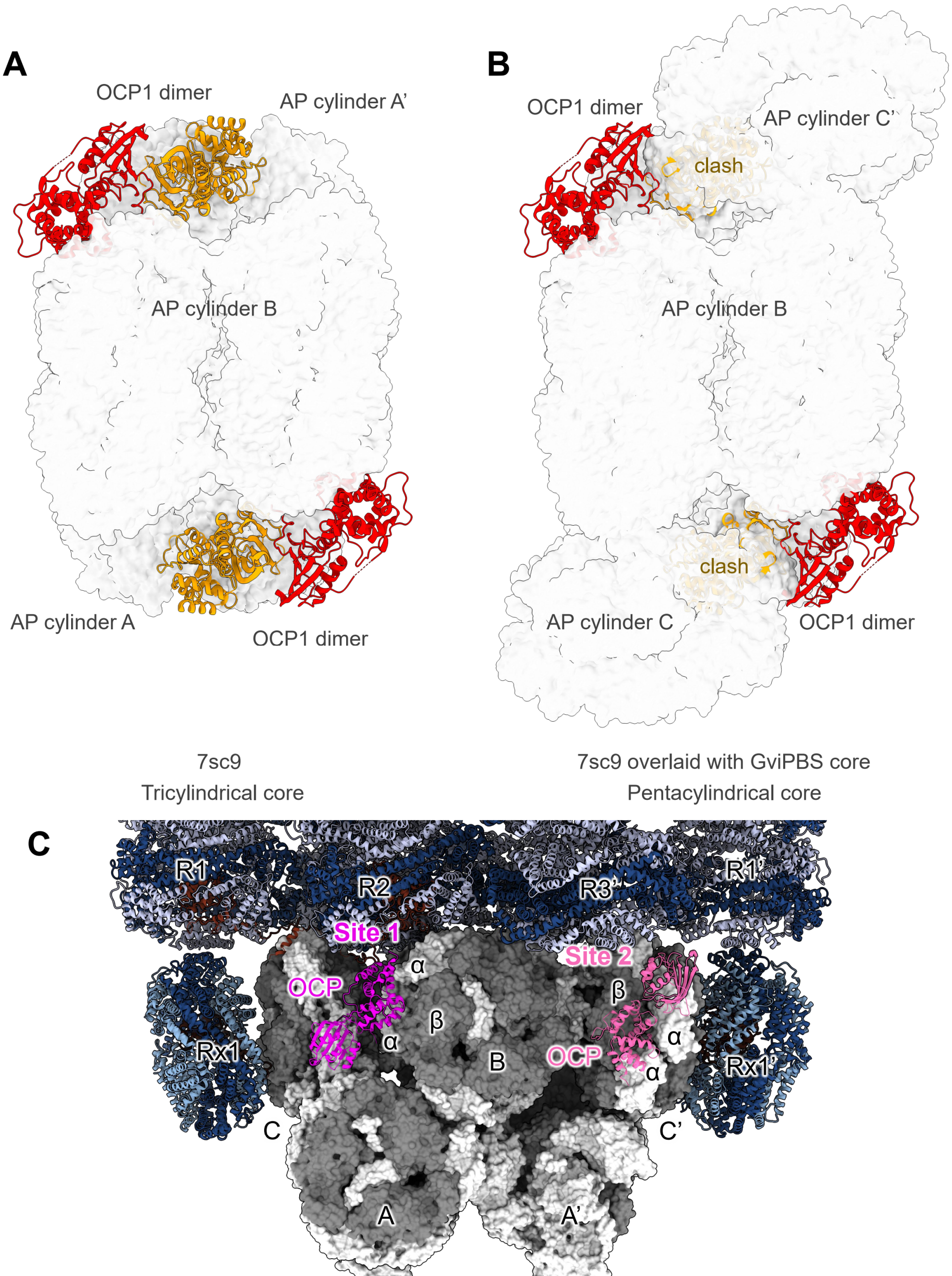

Fig. S15. OCP-binding sites in tri- and pentacylindrical PBS cores. **A**. Cryo-EM structure of OCP1 bound to the tricylindrical core of SynPBS (PDB 7sc9 (*7*)). The AP cores are shown from the top, with the OCP dimer (subunits in red and yellow) bound to the top (B) and bottom (A/A') cylinders. **B**. In the pentacylindrical PBS core, such as in GviPBS, the C/C' cylinders of AP completely mask the OCP-binding site on the A/A' cylinders and thereby block the attachment of the yellow OCP subunit, suggesting an existence of alternative OCP-binding sites. **C**. Structural alignment using conserved AP hexamers and the 7sc9 structure reveals a novel tentative OCP-binding site (Site 2) located on the side of the C/C' cylinder, at the joint of two α and one β AP chains. This joint is equivalent to that of the OCP1-binding site on the top B cylinder observed in the 7sc9 structure (Site 1).

A

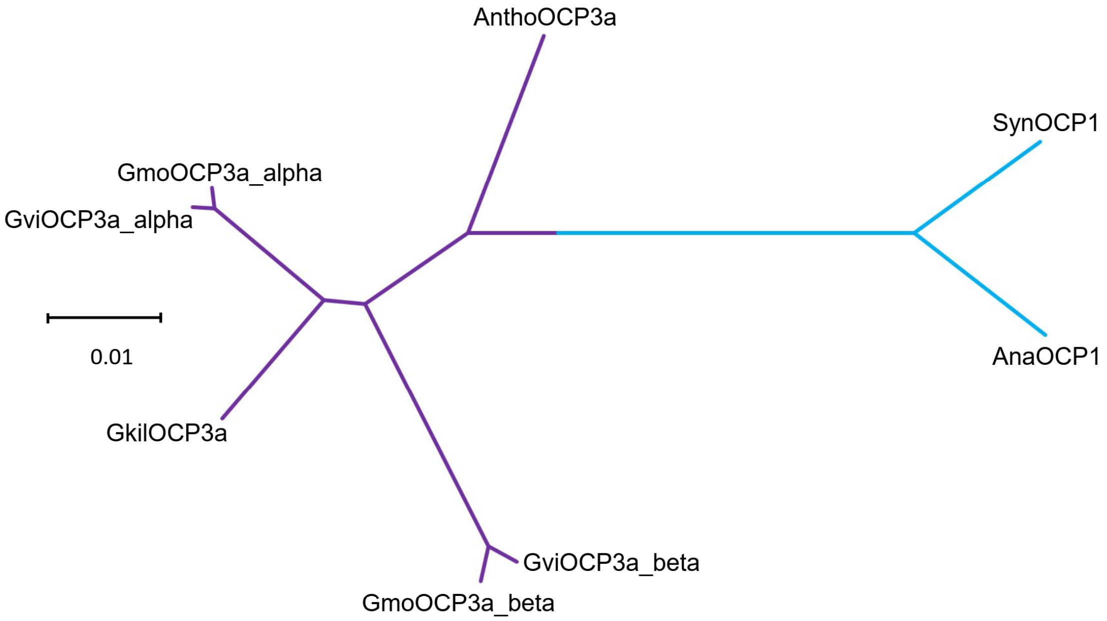

B

SynOCP1	100.00	76.03	47.95	47.32	54.75	52.38	50.79	50.16
AnaOCP1	76.03	100.00	49.69	50.00	52.83	53.63	51.72	51.72
GviOCP3a_beta	47.95	49.69	100.00	94.14	58.93	66.36	66.56	65.94
GmoOCP3a_beta	47.32	50.00	94.14	100.00	57.99	66.04	66.87	66.56
AnthoOCP3a	54.75	52.83	58.93	57.99	100.00	65.52	67.19	65.94
GkilOCP3a	52.38	53.63	66.36	66.04	65.52	100.00	76.71	77.33
GviOCP3a_alpha	50.79	51.72	66.56	66.87	67.19	76.71	100.00	96.30
GmoOCP3a_alpha	50.16	51.72	65.94	66.56	65.94	77.33	96.30	100.00

Fig. S16. Phylogenetic relationships of the OCP3a variants. A. Phylogenetic tree showing distinct clusterization of OCP3a_α and OCP3a_β from different species of *Gloeobacter*, with two OCP1 representatives taken as an outgroup. **B**. Percent identity matrix of the analyzed sequences. SynOCP1 and AnaOCP1 are included for reference. Note that *G. kilaueensis* and *A. panamensis* (AnthoOCP3a) contain single OCP3a variants, of which the former most likely belongs to the α subclade, whereas the identity of AnthoOCP3a is less certain due to its intermediate position. *G. violaceus* and *G. morelensis* contain two OCP3a variants each.

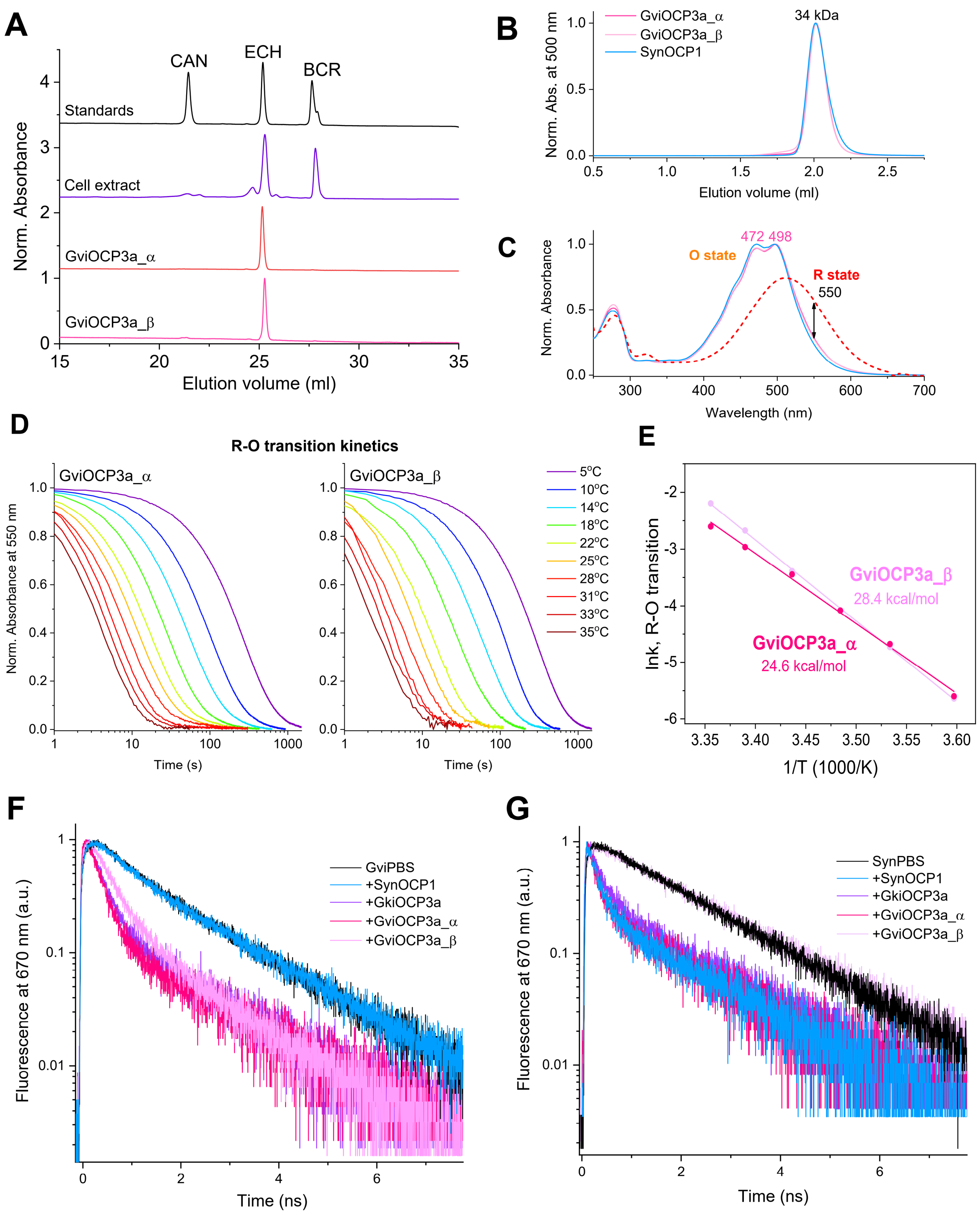

Fig. S17. Characterization of GviOCP3a_α and GviOCP3a_β produced in carotenoid-synthesizing *E. coli* cells. A. HPLC profiles showing that both recombinant proteins bound exclusively echinenone (ECH) under the conditions used for protein expression. No canthaxanthin (CAN) binding was detected as this carotenoid did not have time to form from β-carotene (BCR) and echinenone (ECH) by the crtO ketolase in the expression system used (*35*, *51*). The elution profile for the carotenoid standards is shown. **B,C**. Analysis of recombinant OCP3a variants from *G. violaceus* (GviOCP3a_α and GviOCP3a_β) by size-exclusion chromatography (**B**) and

absorbance spectroscopy (**C**). OCP1 from *Synechocystis* 6803 (SynOCP1) is shown for comparison. The apparent mass of OCP peak is indicated. The absorbance spectra of the dark-adapted orange and photoactivated red states of GviOCP3a_α are shown by solid (O state) and dashed lines (R state). Arrow indicates the wavelength used to monitor the R-O transition. **D**. Characterization of the photoactivity of the GviOCP3a variants by following the R-O relaxation after pre-adaptation to the actinic light (blue LED), at various temperatures as indicated. Note that the proteins exhibit similar photoactivity and relaxation rates in the whole range of temperatures tested. **E**. Arrhenius plot for GviOCP3a_α and GviOCP3a_β characterizing the temperature dependencies of their R-O transition. The activation energy barrier values are indicated. **F,G**. The ability of GviOCP3a variants to quench PBS from *G. violaceus* (**F**) or *Synechocystis* 6803 (**G**) studied by time-resolved fluorescence spectroscopy. The excitation wavelength was 570 nm. SynOCP1 and GkilOCP3a were used as controls. Note that while GviOCP3a_α is capable of quenching both PBS types, GviOCP3a_β is not capable of quenching SynPBS. GviOCP3a_β quenches GviPBS with an altered efficiency, suggesting that GviOCP3a_β binds and quenches GviPBS in an uncommon site.

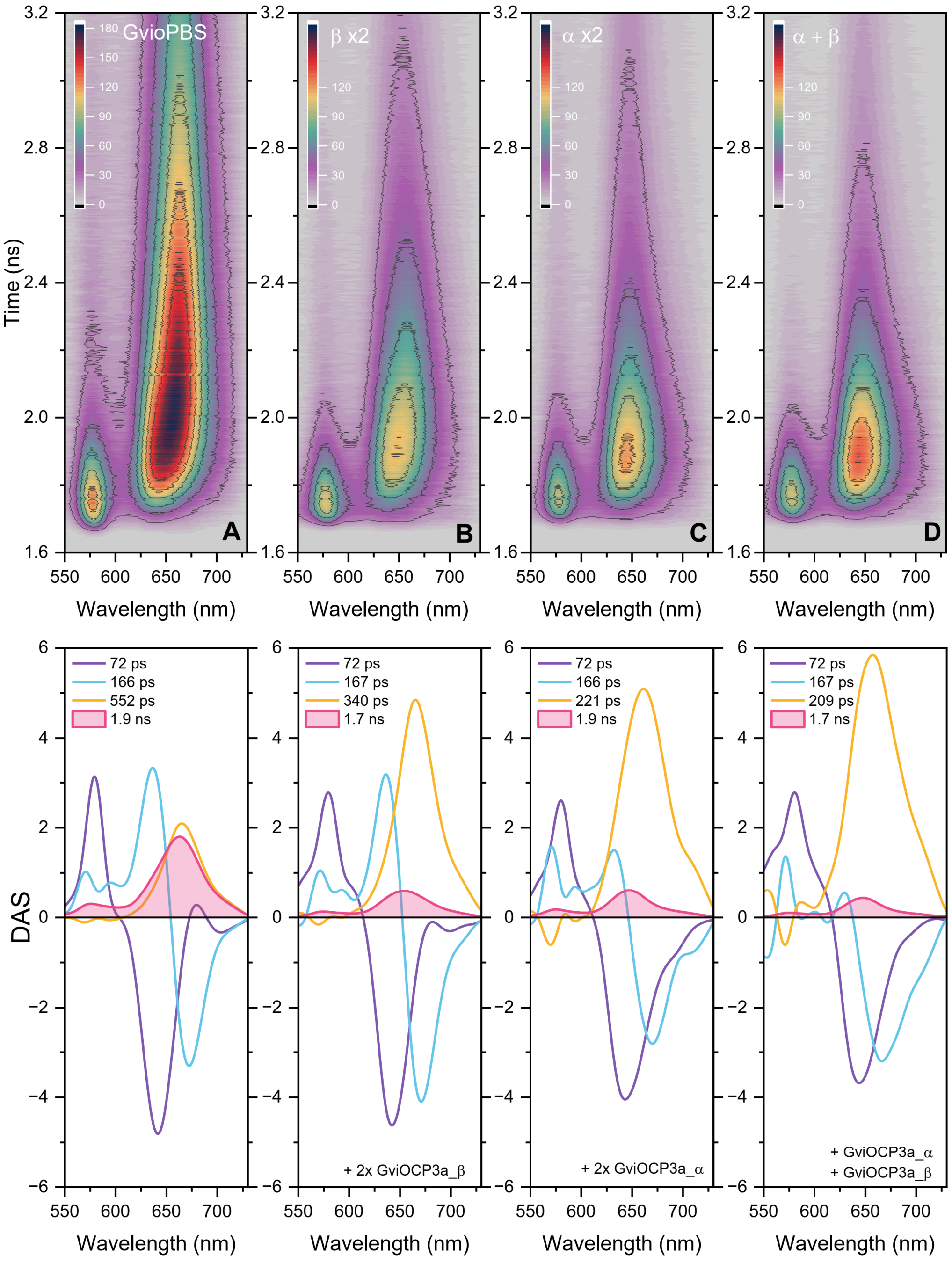

Fig. S18. Study of GviPBS fluorescence by time-resolved emission spectroscopy. Time-resolved emission spectra (TRES) of GviPBS fluorescence in the absence (**A**), presence of the GviOCP3a_β (**B**), GviOCP3a_α (**C**), and their combination (**D**). Below each panel is a corresponding set of Decay associated spectra (DAS) of GviPBS fluorescence. The numbers indicate characteristic kinetic components derived from the global analysis of TRES showing the response to photoactivation of GviOCP3a_β, GviOCP3a_α and their combination. GviPBS fluorescence was excited at 485 nm by 3 mW 150 fs laser pulses at 80 MHz. The temperature of the samples was stabilized at 10 °C during all experiments to prevent fluorescence recovery.

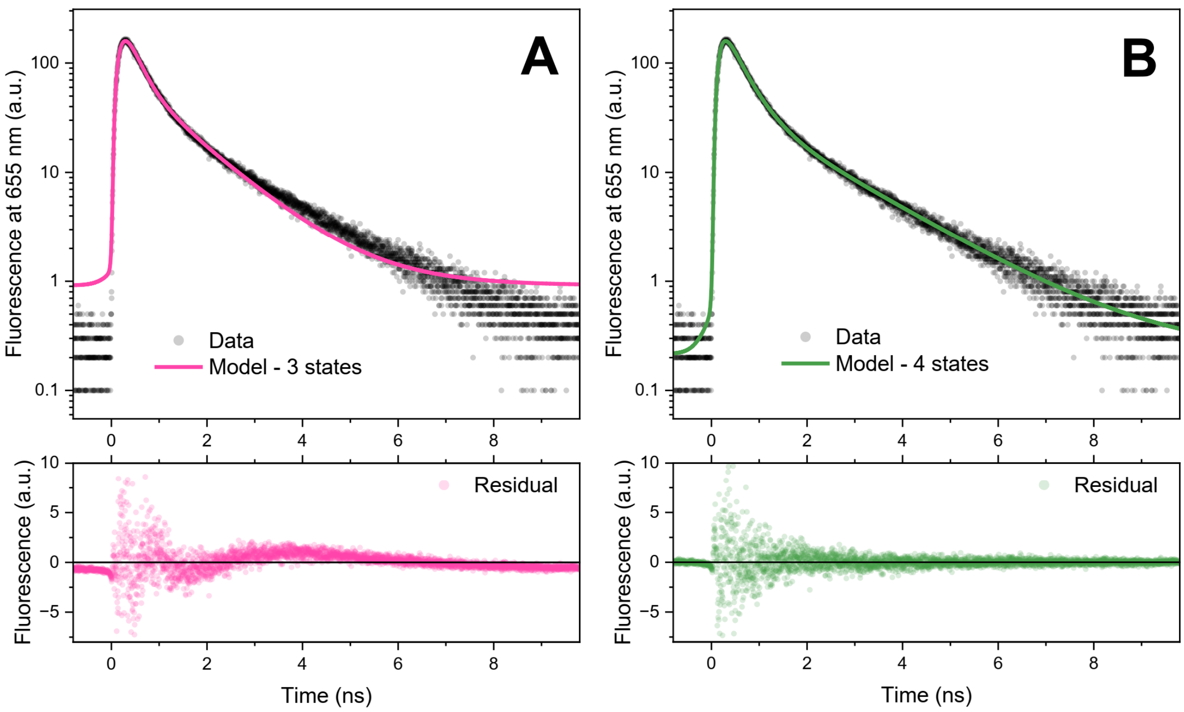

Fig. S19. Typical quality of the fit of an exemplary GviPBS fluorescence decay curve. The experimental curve obtained in the presence of GviOCP3a_α and GviOCP3a_β (semitransparent gray dots) is shown fitted by using three (**A**) or four (**B**) exponentials. Residuals are demonstrated at the bottom. Note that three components give systematic deviations, whereas four components are sufficient for an adequate description of the experimental data.

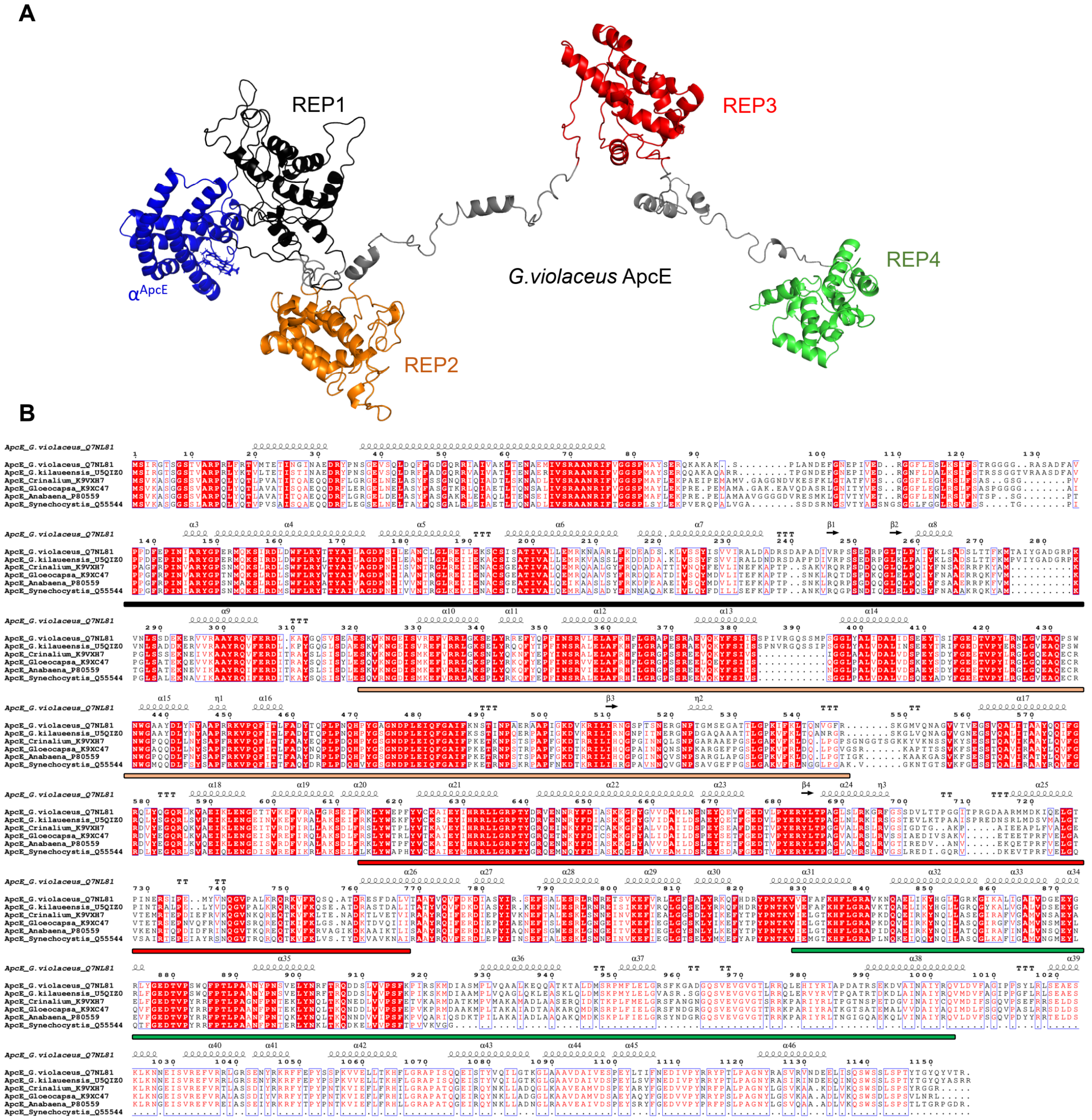

Fig. S20. Domain structure of ApcE proteins. **A**. One copy of ApcE from *G. violaceus* showing the four REP domains following the N-terminal bilin-binding domain α^{ApcE}. **B**. Multiple sequence alignment of ApcE linker proteins from species whose OCP proteins were studied in this work (*G. violaceus*, *G. kilaueensis*, *Crinalium epipsammum* PCC 9333, *Gloeocapsa* sp. PCC 7428, *Anabaena* 29413 and *Synechocystis* 6803). Given that the number of REP domains in ApcE proteins indicate the number of cylinders in the respective PBS core, all taken species possess the pentacylindrical PBS cores (four REP domains), except for *Synechocystis* 6803, which possesses the tricylindrical PBS core (the last REP domain is missing). The location of REP domains are indicated above the alignment with the color coding corresponding to panel A.

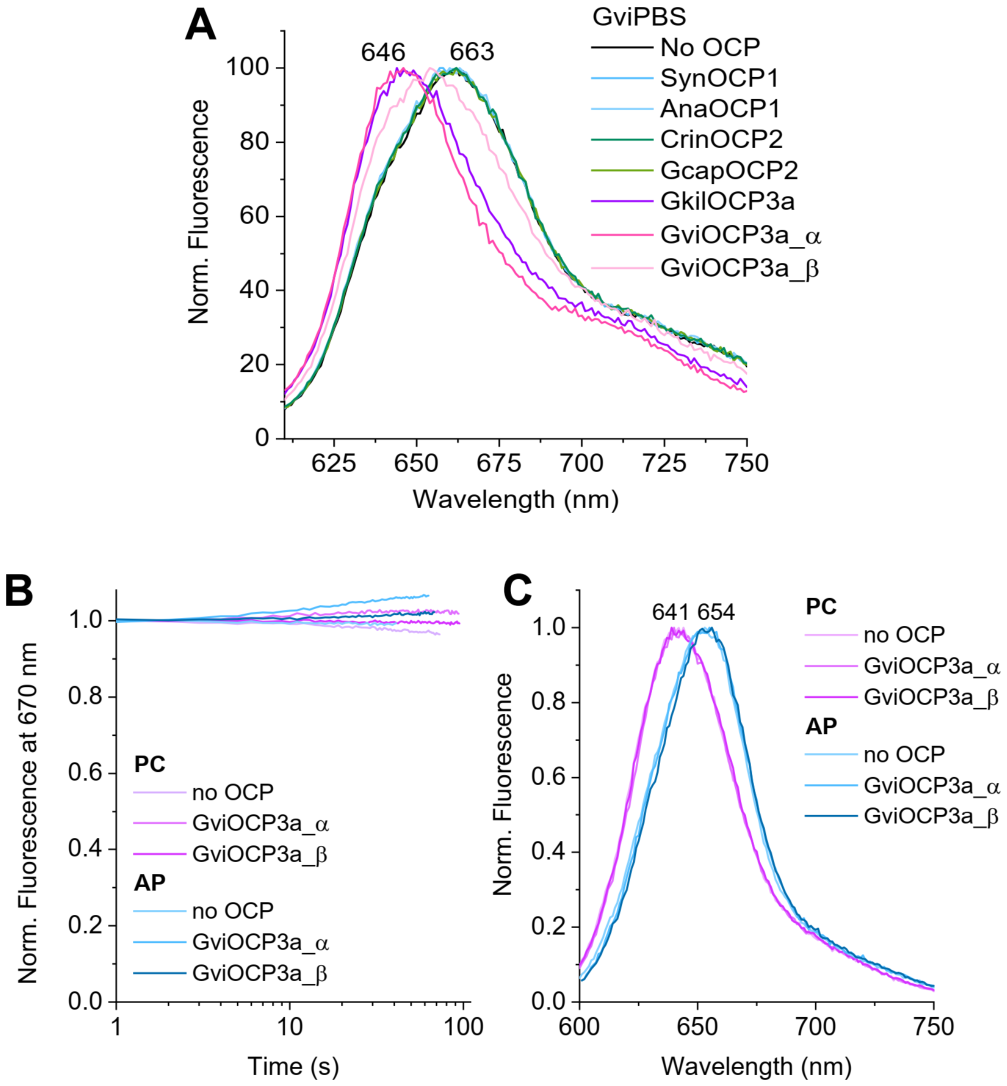

Fig. S21. Effect of GviOCP3a_α and GviOCP3a_β on fluorescence of separated PC and AP components of GviPBS. Temperature 5 °C. **A**. Normalized fluorescence spectra of assembled GviPBS recorded in the absence or in the presence of either OCP variant, as indicated. Note that the maxima of quenched and unquenched GviPBS correspond to those of PC and AP components, respectively. **B**. Changes in the fluorescence intensity of the isolated PC or AP samples over time, with or without the addition of either GviOCP3a variant. **C**. Normalized fluorescence spectra of isolated PC and AP samples recorded in the absence or in the presence of either OCP variant, as indicated. Excitation in all experiments here was at 575 nm.

Table S1.

Mass spectrometry-based identification of PBS proteins from *G. violaceus*. Blue font indicates polypeptides not covered by MALDI MS, but reliably confirmed by LC-MS.

**Protein**	**Category**	**NCBI Reference**	**Uniprot ID**	**Mw, Da**	**Sequence coverage (MALDI)**	**Expect value (MALDI)**	**Sequence coverage (LC-MS)**
ApcA	PBPs	WP_011141246.1	Q7NL80	17537.1	59%	2.6e-004	86.3%
ApcB	PBPs	WP_011141247.1	Q7NL79	17211.7	-	-	91.3%
ApcC	linkers	WP_011141248.1	Q7NL78	7754.0	52%	1.1e-008	52.2%
ApcD	PBPs	WP_011141181.1	Q7NLE3	18032.8	-	-	87.6%
ApcE	PBPs/linkers	WP_011141245.1	Q7NL81	129836.6	57%	2.8e-055	72.1%
ApcF	PBPs	WP_011141928.1	Q7NJA2	17433.9	-	-	80.1%
CpcA	PBPs	WP_011141185.1	Q7M7F7	17662.9	82%	1.4e-005	94.4%
CpcB	PBPs	WP_011141184.1	Q7M7C7	18459.9	79%	2.8e-012	97.7%
CpcC1	linkers	WP_164928640.1	Q7NM19	31043.1	77%	3.5e-014	77.2%
CpcC2	linkers	WP_231848246.1	Q7NGF2	30877.9	65%	6.0e-006	66.2%
CpcD-1	linkers	WP_011141267.1	Q7NL59	7769.9	24%	1.4e-011	90.0%
CpcD-2	linkers	WP_011141266.1	Q7NL60	8169.4	48%	4.5e-007	72.4%
CpeA	PBPs	WP_011141189.1	Q7NLD7	17658.0	59%	3.1e-004	94.5%
CpeB	PBPs	WP_011141190.1	Q7NLD6	18427.2	33%	2.0e-002	83.6%
CpeC	linkers	WP_011141263.1	Q7NL63	31825.2	75%	2.2e-013	87.4%
CpeD	linkers	WP_011141264.1	Q7NL62	28400.2	81%	8.9e-014	67.8%
CpeE	linkers	WP_011141265.1	Q7NL61	28354.9	53%	1.4e-010	66.9%
Glr1262	linkers	WP_011141262.1	Q7NL64	92019.8	53%	5.6e-032	74.9%
Glr2806	linkers	WP_011142800.1	Q7NGT2	81422.5	44%	1.1e-007	43.2%

Table S2.
Cryo-EM data collection, refinement and validation statistics.

	PBS core	PBS C	PBS Rx	PBS R1	PBS R2	PBS R3
PDB	**9V7J**	**9V7K**	**9V7G**	**9V7L**	**9V7H**	**9V7I**
EMDB	EMD-64815	EMD-64816	EMD-64812	EMD-64817	EMD-64813	EMD-64814
**Data collection**						
Grid	Quantifoil R1.2/1.3 300 mesh Cu					
Microscope	Titan Krios					
Energy filter, Camera	BioQuantum, K3					
Energy selecting slit width, eV	20					
Imaging mode	Counting, non-CDS					
Accelerating voltage (kV)	300					
Condenser apertures C1, C2, um	2000, 100					
Spot size	5					
Illumination area, µm	1.1					
Nominal magnification	85,000					
Calibrated pixel size (Å)	0.874					
Total exposure time (sec)	3.9					
Total electron dose ($e^-/Å^2$)	66					
Nominal defocus range (µm)	-0.7 to -1.7					
Number of frames per stack (movie)	60					
Number of stacks (movies) collected	~45,000					
**Data processing**						
Number of stacks (movies) used	41 564					
Original box size (px)	960					
Initial number of particles	1 364 249					
Final number of particles	746,972	746,972	746,972	746,972	207,644	746,972
Pixel size for final map (Å)	1.25	1.25	1.25	1.25	1.25	1.25
Symmetry imposed	C2	C1	C1	C1	C1	C1
Map resolution (Å)	2.85	2.94	2.72	3.76	2.72	3.03
B-factor for sharpening	77.9	70.8	73.8	96.0	60.2	81.4
FSC threshold	0.143	0.143	0.143	0.143	0.143	0.143
**Refinement**						
Initial models (PDB codes)	2VJT, 7EYD	2VJT	2VJR	2VJR	2VJR	2VJR
Model resolution (Å)	2.85	2.94	2.72	3.76	2.72	3.03
Model composition						
Non-hydrogen atoms	123,484	17,887	17,599	36,440	36,476	36,438
Protein residues	15766	2275	2207	4570	4575	4567
Ligands	84	12	12	24	24	24
B factors ($Å^2$)						
Protein	56.7	49.5	41.7	122.9	46.5	56.4
Ligands	51.9	35.3	39.4	112.1	42.5	51.5
R.m.s. deviations						

Bond lengths (Å)	0.01	0.01	0.01	0.01	0.01	0.01
Bond angles (Å)	1.89	1.64	1.58	1.45	1.74	1.69
Validation						
MolProbity score	2.47	2.68	1.67	2.76	2.80	2.82
Clashscore	15.59	7.43	7.43	23.53	21.91	20.20
Poor rotamers (%)	7.27	19.60	2.23	1.75	2.50	3.15
Ramachandran plot						
Favored (%)	83.2	96.9	99.1	81.8	84.9	85.8
Allowed (%)	16.4	2.6	0.8	15.6	12.9	11.6
Disallowed (%)	0.4	0.5	0.1	2.6	2.2	2.6

Movie S1.
GviPBS structure. The cryo-EM reconstruction of GviPBS showing the overall complexity of its architecture, the precise location of the identified linker proteins in the rods and the core, as well as the conformational mobility of the rod bundles sampling conformational space from nearly parallel position to a position diverged by ~15°.

REFERENCES AND NOTES

1. D. A. Bryant, C. J. Gisriel, The structural basis for light harvesting in organisms producing phycobiliproteins. *Plant Cell*, **36**, 4036–4064 (2024).

2. L. Chang, X. Liu, Y. Li, C.-C. Liu, F. Yang, J. Zhao, S.-F. Sui, Structural organization of an intact phycobilisome and its association with photosystem II. *Cell Res.* **25**, 726–737 (2015).

3. X. Zhang, Y. Xiao, X. You, S. Sun, S.-F. Sui, In situ structural determination of cyanobacterial phycobilisome-PSII supercomplex by STAgSPA strategy. *Nat. Commun.* **15**, 7201 (2024).

4. X. You, X. Zhang, J. Cheng, Y. Xiao, J. Ma, S. Sun, X. Zhang, H.-W. Wang, S.-F. Sui, In situ structure of the red algal phycobilisome–PSII–PSI–LHC megacomplex. *Nature* **616**, 199–206 (2023).

5. H.-W. Jiang, H.-Y. Wu, C.-H. Wang, C.-H. Yang, J.-T. Ko, H.-C. Ho, M.-D. Tsai, D. A. Bryant, F.-W. Li, M.-C. Ho, M.-Y. Ho, A structure of the relict phycobilisome from a thylakoid-free cyanobacterium. *Nat. Commun.* **14**, 8009 (2023).

6. Z. Zheng, C. Ma, H. Wang, G. Wang, C. Dong, N. Gao, J. Zhao, The structure of phycobilisome with a bicylindrical core from the cyanobacterium *Synechococcus elongatus* PCC 7942. bioRxiv 650843 [Preprint] (2025). https://doi.org/10.1101/2025.04.28.650843.

7. M. A. Domínguez-Martín, P. V. Sauer, H. Kirst, M. Sutter, D. Bína, B. J. Greber, E. Nogales, T. Polívka, C. A. Kerfeld, Structures of a phycobilisome in light-harvesting and photoprotected states. *Nature* **609**, 835–845 (2022).

8. L. Zheng, Z. Zheng, X. Li, G. Wang, K. Zhang, P. Wei, J. Zhao, N. Gao, Structural insight into the mechanism of energy transfer in cyanobacterial phycobilisomes. *Nat. Commun.* **12**, 5497 (2021).

9. D. A. Bryant, G. Cohen-Bazire, A. N. Glazer, Characterization of the biliproteins of *Gloeobacter violaceus* chromophore content of a cyanobacterial phycoerythrin carrying phycourobilin chromophore. *Arch. Microbiol.* **129**, 190–198 (1981).

10. J. Ma, X. You, S. Sun, X. Wang, S. Qin, S.-F. Sui, Structural basis of energy transfer in *Porphyridium purpureum* phycobilisome. *Nature* **579**, 146–151 (2020).

11. J. Zhang, J. Ma, D. Liu, S. Qin, S. Sun, J. Zhao, S. F. Sui, Structure of phycobilisome from the red alga *Griffithsia pacifica*. *Nature* **551**, 57–63 (2017).

12. G. Guglielmi, G. Cohen-Bazire, D. A. Bryant, The structure of *Gloeobacter violaceus* and its phycobilisomes. *Arch. Microbiol.* **129**, 181–189 (1981).

13. T. Tsuchiya, S. Takaichi, N. Misawa, T. Maoka, H. Miyashita, M. Mimuro, The cyanobacterium *Gloeobacter violaceus* PCC 7421 uses bacterial-type phytoene desaturase in carotenoid biosynthesis. *FEBS Lett.* **579**, 2125–2129 (2005).

14. R. Rippka, J. Waterbury, G. Cohen-Bazire, A cyanobacterium which lacks thylakoids. *Arch. Microbiol.* **100**, 419–436 (1974).

15. B. Kastner, N. Fischer, M. M. Golas, B. Sander, P. Dube, D. Boehringer, K. Hartmuth, J. Deckert, F. Hauer, E. Wolf, H. Uchtenhagen, H. Urlaub, F. Herzog, J. M. Peters, D. Poerschke, R. Lührmann, H. Stark, GraFix: Sample preparation for single-particle electron cryomicroscopy. *Nat. Methods* **5**, 53–55 (2008).

16. H. Wang, Z. Zheng, L. Zheng, Z. Zhang, C. Dong, J. Zhao, Mutagenic analysis of the bundle-shaped phycobilisome from *Gloeobacter violaceus*. *Photosynth. Res.* **158**, 81–90 (2023).

17. K. Kawakami, T. Hamaguchi, Y. Hirose, D. Kosumi, M. Miyata, N. Kamiya, K. Yonekura, Core and rod structures of a thermophilic cyanobacterial light-harvesting phycobilisome. *Nat. Commun.* **13**, 3389 (2022).

18. C. J. Gisriel, G. Shen, G. W. Brudvig, D. A. Bryant, Structure of the antenna complex expressed during far-red light photoacclimation in Synechococcus sp. PCC 7335. *J. Biol. Chem.* **300**, 105590 (2024).

19. E. J. Dodson, J. Ma, M. Suissa Szlejf, N. Maroudas-Sklare, Y. Paltiel, N. Adir, S. Sun, S.-F. Sui, N. Keren, The structural basis for light acclimation in phycobilisome light harvesting systems systems in Porphyridium purpureum. *Commun. Biol.* **6**, 1210 (2023).

20. D. Jallet, M. Gwizdala, D. Kirilovsky, ApcD, ApcF and ApcE are not required for the Orange Carotenoid Protein related phycobilisome fluorescence quenching in the cyanobacterium *Synechocystis* PCC 6803. *Biochim. Biophys. Acta* **1817**, 1418–27 (2012).

21. D. W. Krogmann, B. Pérez-Gómez, E. B. Gutiérrez-Cirlos, A. Chagolla-López, L. González de la Vara, C. Gómez-Lojero, The presence of multidomain linkers determines the bundle-shape structure of the phycobilisome of the cyanobacterium *Gloeobacter violaceus* PCC 7421. *Photosynth. Res.* **93**, 27–43 (2007).

22. E. Berta Gutiérrez-Cirlos, B. Pérez-Gómez, D. W. Krogmann, C. Gómez-Lojero, The phycocyanin-associated rod linker proteins of the phycobilisome of *Gloeobacter violaceus* PCC 7421 contain unusually located rod-capping domains. *Biochim. Biophys. Acta* **1757**, 130–134 (2006).

23. K. Tang, W.-L. Ding, A. Höppner, C. Zhao, L. Zhang, Y. Hontani, J. T. M. Kennis, W. Gärtner, H. Scheer, M. Zhou, K.-H. Zhao, The terminal phycobilisome emitter, LCM: A light-harvesting pigment with a phytochrome chromophore. *Proc. Natl. Acad. Sci. U.S.A.* **112**, 15880–15885 (2015).

24. G. Bernát, U. Schreiber, E. Sendtko, I. N. Stadnichuk, S. Rexroth, M. Rögner, F. Koenig, Unique properties vs. common themes: The atypical cyanobacterium *Gloeobacter violaceus* PCC 7421 is capable of state transitions and blue-light-induced fluorescence quenching. *Plant Cell Physiol.* **53**, 528–542 (2012).

25. A. Wilson, G. Ajlani, J. M. Verbavatz, I. Vass, C. A. Kerfeld, D. Kirilovsky, A soluble carotenoid protein involved in phycobilisome-related energy dissipation in cyanobacteria. *Plant Cell* **18**, 992–1007 (2006).

26. D. Kirilovsky, C. A. Kerfeld, Cyanobacterial photoprotection by the orange carotenoid protein. *Nat. Plants* **2**, 16180 (2016).

27. N. N. Sluchanko, Y. B. Slonimskiy, E. G. Maksimov, Features of protein-protein interactions in the cyanobacterial photoprotection mechanism. *Biochemistry* **82**, 1592–1614 (2017).

28. H. Bao, M. R. Melnicki, E. G. Pawlowski, M. Sutter, M. Agostoni, S. Lechno-Yossef, F. Cai, B. L. Montgomery, C. A. Kerfeld, Additional families of orange carotenoid proteins in the photoprotective system of cyanobacteria. *Nat. Plants* **3**, 17089 (2017).

29. M. R. Melnicki, R. L. Leverenz, M. Sutter, R. Lopez-Igual, A. Wilson, E. G. Pawlowski, F. Perreau, D. Kirilovsky, C. A. Kerfeld, Structure, diversity, and evolution of a new family of soluble carotenoid-binding proteins in cyanobacteria. *Mol. Plant* **9**, 1379–1394 (2016).

30. F. Muzzopappa, A. Wilson, D. Kirilovsky, Interdomain interactions reveal the molecular evolution of the orange carotenoid protein. *Nat. Plants* **5**, 1076–1086 (2019).

31. A. Wilson, C. Punginelli, A. Gall, C. Bonetti, M. Alexandre, J. M. Routaboul, C. A. Kerfeld, R. van Grondelle, B. Robert, J. T. Kennis, D. Kirilovsky, A photoactive carotenoid protein acting as light intensity sensor. *Proc. Natl. Acad. Sci. U.S.A.* **105**, 12075–12080 (2008).

32. M. G. Rakhimberdieva, I. N. Stadnichuk, I. V. Elanskaya, N. V. Karapetyan, Carotenoid-induced quenching of the phycobilisome fluorescence in photosystem II-deficient mutant of *Synechocystis* sp. *FEBS Lett.* **574**, 85–88 (2004).

33. M. Gwizdala, A. Wilson, D. Kirilovsky, In vitro reconstitution of the cyanobacterial photoprotective mechanism mediated by the Orange Carotenoid Protein in *Synechocystis* PCC 6803. *Plant Cell* **23**, 2631–2643 (2011).

34. N. N. Sluchanko, Y. B. Slonimskiy, E. A. Shirshin, M. Moldenhauer, T. Friedrich, E. G. Maksimov, OCP-FRP protein complex topologies suggest a mechanism for controlling high light tolerance in cyanobacteria. *Nat. Commun.* **9**, 3869 (2018).

35. Y. B. Slonimskiy, A. O. Zupnik, L. A. Varfolomeeva, K. M. Boyko, E. G. Maksimov, N. N. Sluchanko, A primordial Orange Carotenoid Protein: Structure, photoswitching activity and evolutionary aspects. *Int. J. Biol. Macromol.* **222**, 167–180 (2022).

36. N. Steube, M. Moldenhauer, P. Weiland, D. Saman, A. Kilb, A. A. Ramírez Rojas, S. G. Garg, D. Schindler, P. L. Graumann, J. L. P. Benesch, G. Bange, T. Friedrich, G. K. A. Hochberg, Fortuitously

compatible protein surfaces primed allosteric control in cyanobacterial photoprotection. *Nat. Ecol. Evol.* **7**, 756–767 (2023).

37. N. N. Sluchanko, E. G. Maksimov, Y. B. Slonimskiy, L. A. Varfolomeeva, A. Y. Bukhanko, N. A. Egorkin, G. V. Tsoraev, M. G. Khrenova, B. Ge, S. Qin, K. M. Boyko, V. O. Popov, Structural framework for the understanding spectroscopic and functional signatures of the cyanobacterial Orange Carotenoid Protein families. *Int. J. Biol. Macromol.* **254**, 127874 (2024).

38. Y. B. Slonimskiy, F. Muzzopappa, E. G. Maksimov, A. Wilson, T. Friedrich, D. Kirilovsky, N. N. Sluchanko, Light-controlled carotenoid transfer between water-soluble proteins related to cyanobacterial photoprotection. *FEBS J.* **286**, 1908–1924 (2019).

39. Y. B. Slonimskiy, E. G. Maksimov, E. P. Lukashev, M. Moldenhauer, T. Friedrich, N. N. Sluchanko, Engineering the photoactive orange carotenoid protein with redox-controllable structural dynamics and photoprotective function. *Biochim. Biophys. Acta Bioenerg.* **1861**, 148174 (2020).

40. V. Krauspe, M. Fahrner, P. Spät, C. Steglich, N. Frankenberg-Dinkel, B. Maček, O. Schilling, W. R. Hess, Discovery of a small protein factor involved in the coordinated degradation of phycobilisomes in cyanobacteria. *Proc. Natl. Acad. Sci. U.S.A.* **118**, e2012277118 (2021).

41. J. Zhao, Y. Weng, Z. Zheng, The structure and mechanism of energy transfer in phycobilisomes. *Annu. Rev. Microbiol.*, 10.1146/annurev-micro-051024-074722 (2025).

42. J. Ma, X. You, S. Sun, S.-F. Sui, Light-induced structural adaptation of the bundle-shaped phycobilisome from thylakoid-lacking cyanobacterium *Gloeobacter violaceus*. *Nat. Commun.* **16**, 5956 (2025).

43. C. L. Grettenberger, Novel *Gloeobacterales* spp. from diverse environments across the globe. *mSphere* **6**, e0006121 (2021).

44. A. Sedoud, R. Lopez-Igual, A. Ur Rehman, A. Wilson, F. Perreau, C. Boulay, I. Vass, A. Krieger-Liszkay, D. Kirilovsky, The cyanobacterial photoactive orange carotenoid protein is an excellent singlet oxygen quencher. *Plant Cell* **26**, 1781–1791 (2014).

45. R. Rippka, J. Deruelles, J. B. Waterbury, M. Herdman, R. Y. Stanier, Generic assignments, strain histories and properties of pure cultures of cyanobacteria. *Microbiology* **111**, 1–61 (1979).

46. D. Tegunov, P. Cramer, Real-time cryo-electron microscopy data preprocessing with Warp. *Nat. Methods* **16**, 1146–1152 (2019).

47. A. Punjani, J. L. Rubinstein, D. J. Fleet, M. A. Brubaker, cryoSPARC: Algorithms for rapid unsupervised cryo-EM structure determination. *Nat. Methods* **14**, 290–296 (2017).

48. E. C. Meng, T. D. Goddard, E. F. Pettersen, G. S. Couch, Z. J. Pearson, J. H. Morris, T. E. Ferrin, UCSF ChimeraX: Tools for structure building and analysis. *Protein Sci.* **32**, e4792 (2023).

49. P. Emsley, B. Lohkamp, W. G. Scott, K. Cowtan, Features and development of Coot. *Acta Crystallogr. D Biol. Crystallogr.* **66**, 486–501 (2010).

50. K. Yamashita, C. M. Palmer, T. Burnley, G. N. Murshudov, Cryo-EM single-particle structure refinement and map calculation using Servalcat. *Acta Crystallogr. D Biol. Crystallogr.* **77**, 1282–1291 (2021).

51. E. G. Maksimov, M. Moldenhauer, E. A. Shirshin, E. A. Parshina, N. N. Sluchanko, K. E. Klementiev, G. V. Tsoraev, N. N. Tavraz, M. Willoweit, F. J. Schmitt, J. Breitenbach, G. Sandmann, V. Z. Paschenko, T. Friedrich, A. B. Rubin, A comparative study of three signaling forms of the orange carotenoid protein. *Photosynth. Res.* **130**, 389–401 (2016).

52. D. V. Zlenko, E. A. Protasova, G. V. Tsoraev, N. N. Sluchanko, D. A. Cherepanov, T. Friedrich, B. Ge, S. Qin, E. G. Maksimov, A. B. Rubin, Anti-stokes fluorescence of phycobilisome and its complex with the orange carotenoid protein. *Biochim. Biophys. Acta Bioenerg.* **1865**, 149014 (2024).

53. Z. J. Jin, Addition in drug combination (author's transl). *Zhongguo Yao Li Xue Bao* **1**, 70–76 (1980).

54. F. Madeira, N. Madhusoodanan, J. Lee, A. Eusebi, A. Niewielska, A. R. N. Tivey, R. Lopez, S. Butcher, The EMBL-EBI Job Dispatcher sequence analysis tools framework in 2024. *Nucleic Acids Res.* **52**, W521–W525 (2024).

55. K. Tamura, G. Stecher, S. Kumar, MEGA11: Molecular evolutionary genetics analysis version 11. *Mol. Biol. Evol.* **38**, 3022–3027 (2021).

56. D. T. Jones, W. R. Taylor, J. M. Thornton, The rapid generation of mutation data matrices from protein sequences. *Comput. Appl. Biosci.* **8**, 275–282 (1992).

57. N. Saitou, M. Nei, The neighbor-joining method: A new method for reconstructing phylogenetic trees. *Mol. Biol. Evol.* **4**, 406–425 (1987).

58. J. Abramson, J. Adler, J. Dunger, R. Evans, T. Green, A. Pritzel, O. Ronneberger, L. Willmore, A. J. Ballard, J. Bambrick, S. W. Bodenstein, D. A. Evans, C.-C. Hung, M. O'Neill, D. Reiman, K. Tunyasuvunakool, Z. Wu, A. Žemgulytė, E. Arvaniti, C. Beattie, O. Bertolli, A. Bridgland, A. Cherepanov, M. Congreve, A. I. Cowen-Rivers, A. Cowie, M. Figurnov, F. B. Fuchs, H. Gladman, R. Jain, Y. A. Khan, C. M. R. Low, K. Perlin, A. Potapenko, P. Savy, S. Singh, A. Stecula, A. Thillaisundaram, C. Tong, S. Yakneen, E. D. Zhong, M. Zielinski, A. Žídek, V. Bapst, P. Kohli, M. Jaderberg, D. Hassabis, J. M. Jumper, Accurate structure prediction of biomolecular interactions with AlphaFold 3. *Nature* **630**, 493–500 (2024).
